# The Transeurope Footrace Project: longitudinal data acquisition in a cluster randomized mobile MRI observational cohort study on 44 endurance runners at a 64-stage 4,486km transcontinental ultramarathon

**DOI:** 10.1186/1741-7015-10-78

**Published:** 2012-07-19

**Authors:** Uwe HW Schütz, Arno Schmidt-Trucksäss, Beat Knechtle, Jürgen Machann, Heike Wiedelbach, Martin Ehrhardt, Wolfgang Freund, Stefan Gröninger, Horst Brunner, Ingo Schulze, Hans-Jürgen Brambs, Christian Billich

**Affiliations:** 1 Department of Diagnostic and Interventional Radiology, University Hospital of Ulm, Germany; 2 Outpatient Rehabilitation Centre at University Hospital of Ulm, Germany; 3 Institute of Exercise and Health Sciences, Sports Medicine, University of Basel, Switzerland; 4 Health Center St. Gallen and Department of General Practice, University Hospital of Zürich, Switzerland; 5 Section on Experimental Radiology, Department of Diagnostic and Interventional Radiology, University Hospital of Tübingen, Germany; 6 Siemens Healthcare, Magnetic Resonance, Stuttgart, Germany; 7 Main organizer and race director TransEurope FootRace 2009, Horb, Germany

## Abstract

**Background:**

The TransEurope FootRace 2009 (TEFR09) was one of the longest transcontinental ultramarathons with an extreme endurance physical load of running nearly 4,500 km in 64 days. The aim of this study was to assess the wide spectrum of adaptive responses in humans regarding the different tissues, organs and functional systems being exposed to such chronic physical endurance load with limited time for regeneration and resulting negative energy balance. A detailed description of the TEFR project and its implemented measuring methods in relation to the hypotheses are presented.

**Methods:**

The most important research tool was a 1.5 Tesla magnetic resonance imaging (MRI) scanner mounted on a mobile unit following the ultra runners from stage to stage each day. Forty-four study volunteers (67% of the participants) were cluster randomized into two groups for MRI measurements (22 subjects each) according to the project protocol with its different research modules: musculoskeletal system, brain and pain perception, cardiovascular system, body composition, and oxidative stress and inflammation. Complementary to the diverse daily mobile MR-measurements on different topics (muscle and joint MRI, T2*-mapping of cartilage, MR-spectroscopy of muscles, functional MRI of the brain, cardiac and vascular cine MRI, whole body MRI) other methods were also used: ice-water pain test, psychometric questionnaires, bioelectrical impedance analysis (BIA), skinfold thickness and limb circumference measurements, daily urine samples, periodic blood samples and electrocardiograms (ECG).

**Results:**

Thirty volunteers (68%) reached the finish line at North Cape. The mean total race speed was 8.35 km/hour. Finishers invested 552 hours in total. The completion rate for planned MRI investigations was more than 95%: 741 MR-examinations with 2,637 MRI sequences (more than 200,000 picture data), 5,720 urine samples, 244 blood samples, 205 ECG, 1,018 BIA, 539 anthropological measurements and 150 psychological questionnaires.

**Conclusions:**

This study demonstrates the feasibility of conducting a trial based centrally on mobile MR-measurements which were performed during ten weeks while crossing an entire continent. This article is the reference for contemporary result reports on the different scientific topics of the TEFR project, which may reveal additional new knowledge on the physiological and pathological processes of the functional systems on the organ, cellular and sub-cellular level at the limits of stress and strain of the human body.

Please see related articles: http://www.biomedcentral.com/1741-7015/10/76 and http://www.biomedcentral.com/1741-7015/10/77

## Background

### Ultramarathon

Various aspects of the physical characteristics of recreational and elite level runners up to marathon distance events have been reported [1-9]. Much less has been written about the anthropometric characteristics of ultra endurance runners [10-14]. The case and field studies of Knechtle *et al*. developed a growing knowledge about the physical characteristics of multistage ultra endurance runners in the past years [15-22]. The German Ultramarathon Association (DUV) defines foot-races of 50 km or longer as ultramarathons (UM). Multistage ultramarathons (MSUM) are races in which each stage has a distance of a UM. Besides a few case reports very little has been reported about the medical aspects of runners doing a transcontinental extended MSUM over several weeks [23]. Until now, there have been no reports published regarding UM running over more than 1,500 km. However, prolonged MSUM races offer the best opportunity to study physical adaptation and the associations of the physiological parameters of athletes in a longitudinal setting day by day.

### The race

**Figure 1:**
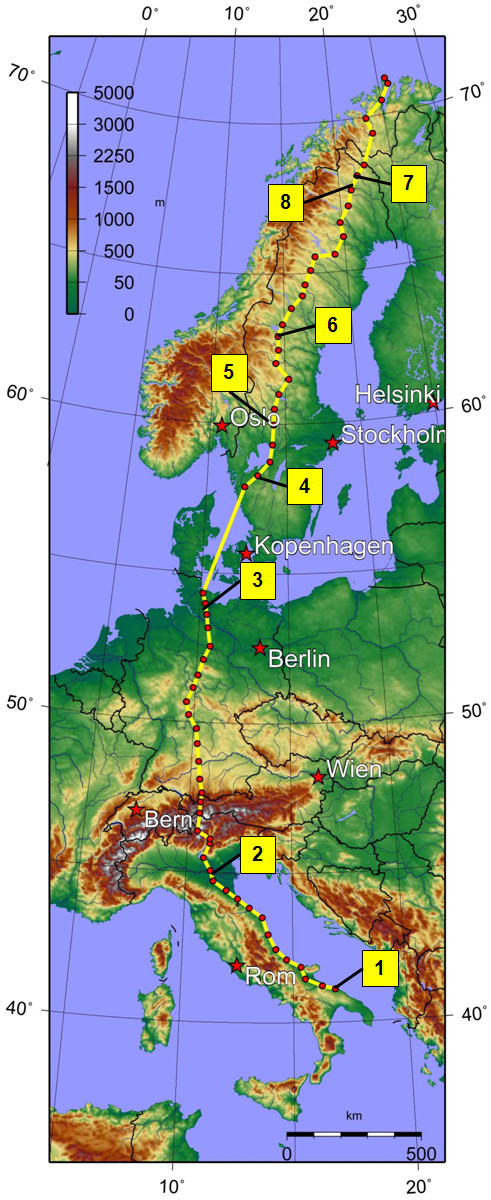
**Route of Trans Europe Foot Race 2009 (4,486 km from south to north of Europe)**.

Among some very heroic solo runs, the TransEurope FootRace 2009 [24] (TEFR09) was the 11^th ^official transcontinental competition multistage footrace within living memory (Table 1) [25-33]. This second European transcontinental MSUM took place from 19 April to 21 June 2009 from Bari, South Italy (41° 8' N, 16° 52' E) to the North Cape, Norway (71°10'N, 25°47'E) (Figure 1). Sixty-seven ultra endurance runners (mean age 50.7 years, range 26 to 74 years, male 56 (83.6%)) from 12 nations (Germany, Japan, Netherlands, France, Switzerland, Norway, Sweden, Finland, Turkey, South Korea, Taiwan, USA) met the challenge and tried to cross six countries (Italy, Austria, Germany, Sweden, Finland, Norway). This comprised running 4,487 km (2,788 miles) in 64 stages without any day of rest. Thus, they expected to complete an average stage distance of 70.1 km, representing 1.7 marathon distances (minimum: 44 km, maximum: 95.1 km) [32].

**Table 1:** History of transcontinental footraces

No	race: date	route	total distance	**days**, stages	mean stage distance	**star **-**ter**^**a**^	**fin**-**isher**^**a**^
**C.C. Pyle's International Trans**-**continental Foot Races (Bunion Derbies 1928, 1929) **[25-27]							

**1**.	1928: 4 March - 24 May	Los Angeles - New York	5,509 km 3,423 miles	84	65.6 km/d 40.8 miles/d	199	55 28%
**2**.	1929: 21 March - 8 June	New York - Los Angeles	5,509 km 3,423 miles	84	65.6 km/d 40.8 miles/d	80	31 39%

**Trans America Foot Races 1992-95 **[28,29]							

**3**.	1992: 20 June - 22 August	Huntington Beach - New York	4,722 km 2,935 miles	64	73.8 km/d 45.9 miles/d	28	13 46%
**4**.	1993: 19 June - Aug.21	Huntington Beach - New York	4,686 km 2,912 miles	64	73.8 km/d 45.9 miles/d	13	6 46%
**5**.	1994: June 18 - 20 August	Huntington Beach - New York	4,708 km 2,926 miles	64	73.6 km/d 45.7 miles/d	14	5 36%
**6**.	1995: 17 June 17 - 19 August	Huntington Beach - New York	4,676 km 2,906 miles	64	73.1 km/d 45.4 miles/d	14	10 71%

**Trans Australia Foot Race 2001**							

**7**.	2001: 6 January - 11 March	Perth - Canberra	4,109 km 2,553 miles	63	67.8 km/d 42.1 miles/d	24	14 58%

**Run Across America 2002**							

**8**.	2002: 15 June - 24 August	New York - Huntington Beach	4,961 km 3,084 miles	71	68.9 km/d 42.8 miles/d	11	8 73%
**10**.	2004: 15 June - 24 August	Huntington Beach - New York	4,961 km 3,084 miles	71	68.9 km/d 42.8 miles/d	10	6 60%

**Trans Europe Foot Races 2003, 2009 **[30-33]							

**9**.	2003: 19 April - 21 June	Lisbon, Portugal - Moscow	5,020 km 3,119 miles	64	79.5 km/d 49.4 miles/d	44	22 50%
**11**.	2009: 19 April - 21 June	Bari, Italy - North Cape	4,486 km 2,787 miles	64	70.1 km/d 43.6 miles/d	67	46 69%

**Run Across America 2011: LANY (Los Angeles to New York)**							

**12**.	2011: 19 June - 27 August	Huntington Beach - New York	5,157 km 3,205 miles	70	73.7 km/d 45.8 miles/d	14	8 57%

^a^total: 528 starters, 224 finishers. 42.4% (some of these have made more than one crossing)

All participants organized their arrival at Bari on their own. Following breakfast at 5:00 a.m., the daily stage started at 6:00 a.m. The race director, together with his staff, planned the stages with their corresponding distances and ascent or descent and organized the accommodations for the runners in halls as well as the food for each stage. In addition, most of the runners carried individual nutrition on their own. Depending on the stage length, five to ten stop points for nutrition were placed on the daily routes. After each stage the runners had time on their own (nutrition, sleeping, regeneration). Depending on the stage length and local situation, dinner was served between 5:00 and 9:00 p.m. The runners slept in camping grounds (mainly in Italy), local sport halls or local community halls at the stage destinations (9:00 p.m. to 4:00 a.m.). Sometimes the quarters were crowded resulting in difficult sleeping conditions. In total, the runners had about 7 to 13 hours of rest per day for recuperation. The local sanitary conditions also changed daily and from country to country.

### The project

The TEFR09 project is the first observational cohort study intended to produce unique and comprehensive data from longitudinal measurements of a large sample of ultra endurance runners taking part in one of the most extreme multistage endurance competitions in the world, which takes the participants to a different level, where the race becomes a way of life, and where nutrition, sleep, energy and psychological states have to be carefully managed [34,35]. It is also the first study using a mobile MRI scanner for continuous examination of the athletes while performing a transcontinental MSUM. The aim was to explain the wide spectrum of adaptive responses in humans being exposed to such a chronic physical endurance load with negative energy balancing but without enough time for regeneration and to identify factors associated with inter-individual variation in these responses. Due to the unique possibility to observe morphological and physiological changes and the reactions of different tissues and functional systems on the systemic, organ and (sub)cellular level with modern MRI techniques, a wide range of multiple questions, hypotheses and unproven assumptions regarding injury, adaptation, regeneration, reparation and overuse processes arose. Therefore, four different project modules of investigations were created, focusing on multiple open questions and unproven hypotheses regarding long distance running:

### Project module I: musculoskeletal system

#### Pre-race injuries and deformation

It is inevitable that pre-race injuries or biomechanical deviations from the norm (for example, mal-alignment) will lead to progressing focal damage of the lower extremities when performing a transcontinental MSUM. If any participant who suffers from unhealed pre-race injuries or deformation of the lower extremities reaches the finish line at North Cape without deterioration, this hypothesis can be rejected. Furthermore, the TEFR project tries to detect reasons for not finishing the race in detail.

#### Joints

Recent investigations indicate that running a marathon does not increase pathologies of structures of the knee joint [36-39]. Some authors postulate a risk factor of repeated marathon running for osteoarthritis of the knee [40]. However, a protective effect of running for knee joint cartilage is also discussed [41,42]. Nothing is known about the effect of ultra long distance running over weeks as in MSUM on knee structures. For the knee cartilage, experimental studies on animals using quantitative microspectrophotometry and polarized light microscopy showed a decrease of the glycosaminoglycan content of the superficial femorotibial joint cartilage layer and degradation and reorganization of the superficial collagen network [43-45]. With this study, there are two hypotheses to be proven: first, in a MSUM over nine weeks, the well-trained participants show no increase in pathologies of the structures of the knee joint; and second, the cartilage of the femorotibial joint shows a degradation of the glycosaminoglycan content with quick regeneration after the end of the race. The hypothesis that MSUM running does not indicate a higher risk of osteoarthritis of the knee in well-trained endurance runners has to be proven with the TEFR project. Contrary to the femorotibial joint the following hypothesis for the femoropatellar joint has to be proven: retropatellar cartilage degeneration is not caused by MSUM running. The femoropatellar joint is not a limiting factor for ultra endurance running, although degeneration arthrosis is already present in some MSUM participating athletes, respectively. This hypothesis is based on the fact that retropatellar arthrosis has a high prevalence in older people [46] and long distance running participation rises with age [47]. This TEFR project will also prove the same hypothesis for the joints of the ankle and hindfoot. For the latter no specific studies have been published until now.

#### Soft tissues of the leg

The literature on long distance running demonstrates that injuries mostly occur in the active and passive soft tissues of the lower extremities [48-54]. Injuries of the muscles, tendons and fascia of the lower legs are the most obvious limiting factors for performance and the most common reasons for not finishing a transcontinental footrace. This postulation leads to the following hypothesis which has to be proven by the TEFR project: During a MSUM every participant will suffer from different injuries and overload of active and passive soft tissues of the lower musculoskeletal system, resulting in long lasting damage due to lack of recreation time.

A special problem for endurance runners is a pain syndrome of the lower leg, so-called 'shin splint'. Investigations over the past years, especially with MRI, showed that several entities caused by overuse of the lower leg due to long distance running can be differentiated. Some authors indicated that bone or periosteal reactions of the ventral tibia are a typical part of this syndrome [55,56], while others do not consider this as being mandatory [57]. Depending on the involved tissues and the lack of exact knowledge of the pathogenesis, the terminology regarding chronic lower leg pain in runners is broad and has not been differentiated and defined in detail until now: medial tibial stress syndrome, shin splint, anterior muscle syndrome, (peri-)myositis, periostitis, fasciitis, and so on [58,59]. With our study design we try to prove the hypothesis that running-associated chronic lower leg pain includes different entities, such as overuse pathologies of muscles, fascias, tendons and bone tissues of the lower leg. The problem begins in the friction areas of the fascia of the muscles and the tendons (peritendineum) and then extends to other tissues such as the muscles, periosteum and bone if the running burden continues and the pain is ignored by the athlete. As many ultra athletes reported, it seems to be possible to overrun 'shin splint' without further damage. Whether medial tibial stress syndrome can end up in a stress fracture or a chronic exertional compartment syndrome when running is continued without any further rest is not clear [56,60,61]. Perhaps further understanding and differentiation of soft tissue overuse of the leg is possible due to this observational cohort study with a mobile MRI.

#### Bones

Stress fractures of the lower body often occur in people doing extensive walking or running without proper training or adaption to the repeated and persistent mechanical burden their bones have to deal with. As seen in young soldiers doing their first long march with full body equipment [62] or amateur runners or beginners in (half-) marathons [63] our hypothesis is that even well trained ultra runners, such as the TEFR participants, can suffer stress fractures, because their skeletal system is not adapted to this tremendous mechanical burden that occurs while crossing a continent by foot with a speed of more than 6 km/hour without any day of rest. The TEFR project with mobile MRI tries to detect early signs of bone reaction (for example, subperiosteal new bone formation, adaptive cortical hypertrophy with little true inflammation, local bone edema or bruising) indicating overload as precursors of stress fractures [59].

For asymptomatic healthy marathon runners significantly higher red bone marrow hyperplasia has been observed compared to healthy volunteers [64]. This is postulated to be a response to 'sports anemia', which is commonly found in highly conditioned trained athletes. The TEFR project wanted to prove the hypothesis that during a MSUM lasting more than nine weeks an increase of red bone marrow occurs in the participants, even though they are adapted and well trained in ultra running.

### Project module II: brain, mind and pain perception

#### Brain volume

Aerobic exercise protects from insular atrophy in the brain of healthy volunteers [65,66]. The normal annual volume loss due to age-related brain atrophy is about 0.11% [66,67]. Increased atrophy is shown for several diseases, such as Alzheimers (2% per year) [68,69] anorexia nervosa [70,71] or malnutrition based on other reasons [72]. Some hypotheses explain this based on the influence of the stress hormone cortisol [70] but further pathophysiological principles leading to this decrease in brain volume are not understood [73-76]. Marathon-induced changes in endocrine levels [77,78], in fluid balance [79] and amino acid blood level [80] are known to alter brain metabolism. The hypothesis that ultra endurance running leads to volume reduction of the brain cortex could be investigated by the TEFR project using high resolution cerebral MRI. Combined with specific laboratory analyses of different markers (for example S100B [81,82]) in blood and urine, reasons explaining the mechanism may be identified. On the other hand, when we postulate that an ultra long MSUM, such as the TEFR09, modifies the plastic brain (state marker) the hypothesis that the sensomotoric cortex volume - which is responsible for the lower extremities - will increase has also to be proven [83].

#### Brain lesions

Additionally we hypothesize that the well-known exercise-induced hyponatremia due to inappropriate arginine vasopressin secretion which can lead to encephalopathy [84-87] is not seen in the highly endurance-trained participants of the TEFR09. If an MSUM leads to brain lesions, water sensitive cerebral MRI sequences of the TEFR project will show it.

#### Pain perception, mind and mental stress

All participants in the TEFR09 had previously finished an ultra marathon. This unique collective of endurance athletes is eminently suitable for examining the hypothesis that ultra runners have different mental prerequisites (higher auto suggestibility) compared to the normal population (trait marker). Experienced ultra endurance runners often mentioned that finishing an ultra race is more a matter of mind than a matter of the body. The hypothesis that finishers of MSUM differ from non-finishers with regard to pain suppression and willpower can be proven by pain tests combined with functional MRI of the brain and mental stress markers in serum samples.

### Project module III: cardiovascular system

#### Heart

The MSUM TEFR09 results in an extreme prolonged stress for the whole organism. Its effects on the heart can be discussed controversially. Cardiac dysfunction after marathon running is verified with biochemical markers and cardiac ultrasound [88,89]. Investigations with cardiac MRI are inconsistent; cardiac damage such as myocardial necrosis is seen in middle-aged marathon runners (57.2 +/- 5.7 years) [90], but not in younger marathon participants (30 to 50 years) [91,92]. Myocardial function disorders are reported with cardiac MRI tagging [93]. Until now, diagnostic analyses of long-lasting running effects on the heart using cardiac biomarkers are difficult to interpret, for example, the increase in brain natriuretic peptide (BNP) after 100 km trials [94,95] could show cytoprotective or growth regulatory effects [89,96] but also myocardial insufficiency. The TEFR project intends to prove the following hypotheses: Even in well trained ultra endurance runners, the running burden of a transcontinental MSUM of more than nine weeks induces a progressive cardiac distress and signs of cardiovascular mal-adaption. Cardiac MRI, stress laboratory tests and ECG might show signs of cardiac structural and functional restrictions, dysfunctions, damages and insufficiency. Non-finishers of the TEFR09 will show more restrictive parameters than finishers. Another hypothesis contradicting the Morganroth hypothesis [97,98] is proven: Although an aerobic endurance burden is performed, the TEFR09 finisher will show an increase in cardiac output and left ventricular mass index and in ventricular wall thickness, which might be caused by incomplete or critical left ventricular hypertrophy in some cases. Using a MR tagging technique, we will try to prove the hypothesis that the anatomical position of the heart is going to change (steep position) with a prolonged aerobic running burden [99].

#### Arteries

Arteries of the muscular-type, such as the common femoral artery (CFA) adapt structure and function to endurance exercise training and elastic-type arteries, such as the common carotid artery, show functional adaptations to increased endurance exercise training. Schmid-Trucksäss *et al*. [100] found an increase of diameter and compliance and a constant shear rate for CFA using noninvasive vascular ultrasound in highly endurance-trained athletes compared to sedentary controls. Other authors postulate that endurance exercise does change arterial wall stiffness and vascular (endothelial) function [101-103]. The TEFR project tries to prove the hypotheses that the MSUM burden results in cardiovascular adaptations in the form of an increase of arterial wall compliance in the lower extremities associated with an increase in the vessel lumen diameter of the femoral artery and of the central aortal pulse wave velocity even in well-trained ultra-endurance runners. This will result in a decrease of the peripheral arterial resistance leading to an increase in basic perfusion of the lower extremities.

### Project module IV: body composition

Endurance exercise leads to a reduction of subcutaneous fatty tissue as demonstrated in several field studies [104-107]. It is well known that fat is the main energy-rich substrate for ultra endurance performance [105,107,108]. In contrast, muscle tissue provides lower energy, when being catabolized. A decrease in skeletal muscle mass due to ultra endurance performance has only been demonstrated in case reports [15,23,109] or small series [108].

Different effects for long-lasting or ultra endurance performances on body composition are described in the literature and seem to depend on the type of endurance burden. In ultra endurance performance with defined breaks (for example, during the night), body mass may remain stable [110-112] or even increase [105] while body fat is reduced [104,105,113], whereas skeletal muscle mass seems to be spared [111,113,114] or may even increase [104]. Ultra endurance performance over hours, days or weeks without a break, results in a decrease in body mass [23,107,109,115] in which body fat as well as skeletal muscle seems to decrease as a few case reports indicate [15,23,109]. With this cohort study we can prove the hypothesis that due to the immense negative energy balance during a transcontinental MSUM, not only fat but also lean tissue is involved in catabolism, even in the leg muscles. With its mobile whole body MRI protocol, the TEFR project will be able to measure the different amount of mass loss in the different functional muscle units of the leg. We also intend to detect the microstructural and intracellular adaption processes of leg muscle tissue with modern MRI methods (MR-spectroscopy, diffusion and perfusion MR imaging).

### Purpose

This report describes the design and conduct of the TEFR project. We report the pattern of chronic endurance running exposure, characteristics of the subject groups and reasons for not finishing. Detailed descriptions of measuring methods in relation to the hypotheses and technical challenges encountered in the realization of the interdisciplinary TEFR project are presented. We discuss the strengths and limitations of the study setting.

## Methods

After commitment to funding by the German Research Society (DFG) the 67 TEFR09 participants were asked to join the TEFR project, which was approved by the local ethics committee of the University Hospital of Ulm (UHU, No.: 270/08-UBB/se), Germany (in accordance with the Declaration of Helsinki) regarding the study design, risk management plan and individual protocols. Verbal and written informed consent was obtained from all concurring subjects.

### Mobile MRI

**Figure 2:**
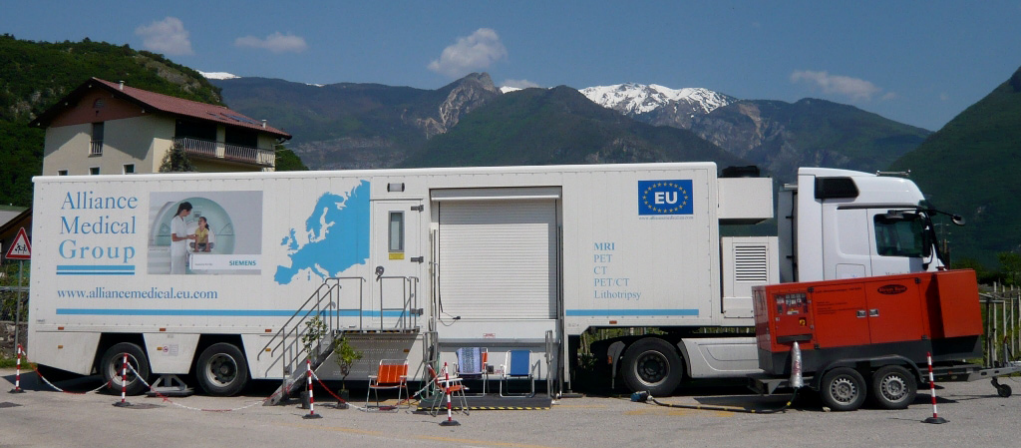
**Truck trailer with mobile MRI and external generator in working position**.

The most important research tool was a 1.5 Tesla whole-body MR imager (Magnetom Avanto™ mobile MRI 02.05, software version: Syngo™ MR B15, Siemens Ltd., Erlangen, Germany) mounted on a mobile unit (MRI-Trailer Model Mob.MRI 02.05, SMIT Mobile Equipment B.V., Division AK Specialty Vehicles, Farnham, UK) pulled by a specially hired truck tractor. The semi-trailer had an internal diesel generator to power the helium cooling circuit for the MRI over the ten-week period. However, it did not generate enough electricity for continuous MRI measurements and was therefore supplemented by a more powerful custom made external diesel generator (150 KVA, Strom Rent™ e.k., Dortmund, Germany) which was pulled by an additional material van. The mobile hardware had a total weight of more than 45 tonnes and was nearly 30 meters long. All of the equipment was installed daily at each stopover and required daily checks and support of all technical systems (Figure 2).

### Study participants

Forty-four (67%) of the race participants (mean age 49.7 years, range 26 to 68 years, male 40 (90.9%), f 4) were recruited for the TEFR project. The inclusion criterion obviously was an official acceptance as a participant at the TEFR09 by the organizers and the race director. The conditions of participation were: minimum age 18 years, the presence of a medical certificate not older than 30 days which indicated physical health and clear proof of appropriate running performance in the field of UM. The specific running history and performance of the individual subjects can be described by different traits, which were requested before the start of the TEFR09: years of regular endurance running training, finished (ultra-) marathons, personal best times in different defined ultra races and extent of training (volume, duration, intensity) before TEFR09.

The investigators additionally performed a resting cardiovascular check using a 12-channel PC-ECG system (Custo cardio 100™, Custo Med Ltd., Ottobrunn, Germany) and blood pressure (RR) measurement using a manual sphygmomanometer (BOSO Clinicus, JungingenGermany: to the nearest 3 mmHg). Cardiovascular exclusion criteria were resting blood pressure > 200 mmHg systolic and/or > 110 mmHg diastolic, acute systemic infection, acute chest pain and new arrhythmias or ECG changes. An orthopedic physical examination was done focusing on contraindications for endurance running such as relevant malalignment and painful joint diseases of the lower extremities. Additional specific exclusion criteria were contraindications against MRI scanning (for example, metallic foreign bodies in dangerous locations, specific cochlear or ocular implants, ferromagnetic vascular clips and relevant claustrophobia). None of the volunteers had to be excluded from study participation due to these criteria.

### Investigators

Four members of the TEFR project comprised the investigator core team that accompanied the TEFR09 for direct data acquisition before and during the race: two physicians, one medical student and one radiological assistant. The latter (HW) was responsible for subject positioning in the scanner and performance of the MR examinations. One of the investigators, the initiator and main organizer of the TEFR project (US), drove the MR-trailer truck, adapted the daily research program to the actual circumstances, controlled and checked the quality of the MR examinations and was responsible for the technical readiness of the whole mobile MRI and its functional circuits and equipment with external and internal diesel generator. Being specialized in radiology and orthopedic surgery, he also did the initial and follow-up physical musculoskeletal examinations of the subjects. The second investigator (CB) was responsible for acquisition of daily anthropometric, laboratory and ECG data. The medical student (ME) made the daily anthropological measurements.

The two physicians were solely responsible for the study and gave neither training advice nor provided medical help.

### Study design

**Figure 3:**
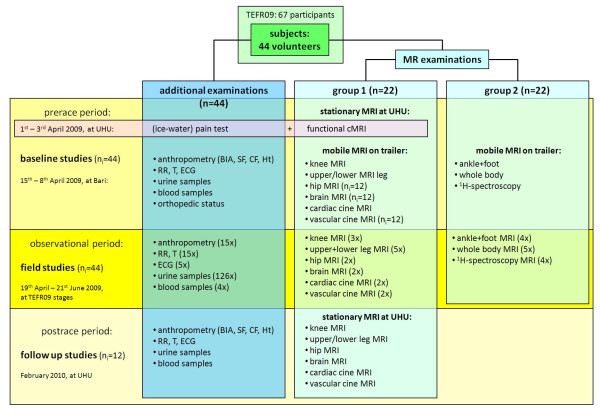
**Study design of TEFR-project**.

The study design of the TEFR project is shown in Figure 3.

### Pre-race

Baseline studies were performed within the last four days before the start of the TEFR09 in Bari on every subject. They included group specific MRI examinations and anthropometric and cardiovascular physical measurements with urine and venous blood samples.

Additionally, body height measuring using a wall-mounted stadiometer (to the nearest 5 mm, standing barefoot) and active range of motion measurement (AROM) of hip and knee joints using a manual double-armed universal goniometer (to the nearest 5°) were done before the start. One experienced orthopedic surgeon, trained in a standardized procedure for positioning both the subject and the goniometer, collected these data.

Adapted from the methods of Paley *et al*. [116] and Weidelich *et al*. [117], analysis of lower limb alignment was done on coronal lower body scout views of pre-race MRI with subjects in the supine position and with extended legs. Measured parameters were: leg length (LL), as the straight line from the middle of the femoral head to the midpoint of the upper talus rim; femorotibial angle (FTA), as the angle between anatomical femoral and tibial axis; the mechanical axis deviation (MAD) as the distance from the point of intersection between the perpendicular and mechanical axis of the limb (straight line from the middle of the femoral head to the midpoint of the upper talus rim) to the midpoint of the knee (medial tibial eminence) and the femoral to tibial length ratio (F/T) [116-122].

A 240-item, 31-dimensional personality temperament and character inventory (TCI) [123,124] in addition to a 10-item, 4-scaled questionnaire on self expectancy (General Self-Efficacy Scale, GSE) [125,126] were also integrated into the project before its start.

Additionally, 15 of the 44 subjects had an initial separate pre-race test on pain perception (ice-water test) combined with functional cerebral MRI two weeks before the start of the TEFR09 at UHU on 1 to 3 April 2009, because these examinations could not be implemented on the mobile MRI due to technical limitations. Due to the exorbitant physical and mental burden placed on the subjects, there was no opportunity for field experiments, invasive tests or application of psychometric instruments during the transcontinental foot-race.

### Field studies

The observant field studies during the TEFR09 were completed between 15 April and 21 June 2009. Every morning from 3:45 to 4:30 a.m. urine samples and anthropometric measurements were taken. The core team broke down their examination units and drove to the next stage destination. Before stage length dependent arrival of the first runner they had set their systems ready and had refuelled the generators and vehicles. MRI examination time was between 2:30 p.m. +/- 90 minutes and 9:00 p.m.). At the same time anthropometric and cardiovascular physical measurements, blood and urine samples were collected and ECG was done. The daily data acquisition also included measurement and documentation of daily weather conditions (temperature outside and inside, humidity outside) using a calibrated electronic thermometer, the stage length and the individual stage performances of the runners (stage running time).

### MR measurements

For MR measurements two groups (22 subjects each) were cluster randomized according to the different research modules. The MR protocols were created in an interdisciplinary content ensuring multifold specific and diverse but precise analyses and measurements for detailed testing of the mentioned hypotheses concerning long distance running (Table 2).

**Table 2:** MRI protocols of the TEFR project

**MRI in module I: Musculoskeletal System**

**ankle/foot **(PP: FFS, supine):
• **TIRM **2D sag; **PM**: FA 140, TE 60; TR 11320, IR 120, ST 2, SBS 2.4, FOV 900, MS 512*512, PS 0.586 iso, PB 130; **IAT**: 5:37
• **PDw TSE fs **2D tra; **PM**: FA 150, TE 32, TR 5830, ST 4, SBS 4.4, FOV 256, MS 384*384, PS 0.4167 iso, PB: 150; **IAT**: 3:46
• **syngo™ MapIt FLASH **2D sag: T2*w GRE; **PM**: FA 60, TE 4.5, TR 1010, ST 2.5, SBS 2.75, FOV 182.25, MS 320*320; PS 0.421875 iso, PB: 250, IN: 12; **IAT**: 4:15

**knee **(PP: FFS, supine):
• **TIRM **2D sag; **PM**: FA 140, TE 50, TR: 4010, TI 150, ST 3, SBS 3.3, FOV 289, MS 256*256, PS 0.664 iso, PB 180; **IAT**: 3:31
• **PDw TSE fs **2D cor; **PM**: FA 150, TE 31, TR 4400, ST 3, SBS 3.3, FOV 289, MS 512*512, PS 0.332 iso, PB 100; **IAT**: 4:11
• **syngo™ MapIt FLASH **2D tra/sag: T2*w GRE; **PM**: FA 60, TE 4.18, TR 889/1120, ST 3, SBS 3.3, FOV 289, MS 512*512; PS 0.332 iso, PB: 250, IN: 12; **IAT**: 4:21/3:58

**Hip **(PP: FFS):
• **TIRM **2D cor; PM: FA 150, TE 61, TR 6230, TI 145, ST 3.5, SBS 3.85, FOV 1444, MS 384*384, PS 0.9896 iso, PB 130; **IAT**: 03:38

**upper/lower leg **(PP: FFS, supine):
• **T1w SE **2D tra, **PM**: FA 90, TE 13, TR 626, ST 5, SBS 5, FOV 1050/722, MS 512*336/256, PS 0.78125/0.7422 iso, PB 115; **IAT**: 1:54/1:30
• **TIRM **2D tra; **PM**: FA 140, TE 62; TR 12530, TI 130, ST 3, SBS 3.9, FOV 512, MS 384*192, PS 0.833 iso, PB 180; **IAT**: 2:08
• **PDw TSE fs **2D tra; **PM**: FA 150, TE 39, TR 6730, ST 3, SBS 3.9, FOV 512, MS 320*160, PS 1.0 iso, PB: 150; **IAT**: 2:14
• **DWI **(with **ADC**): **SPAIR **epi b-value 0-800, 2D tra; **PM**: FA 90, TE 75, TR 5100, ST 10, SBS 10, FOV 1300/1173.25, MS 128*104, PS 3.125/2.96875 iso, PB 1030; **IAT**: 2:32/3:01

**MRI in module II: Brain and Pain**

**Brain **(PP: HFS, supine):
• **(turbo) FLASH 3D **sag: T1 mpr; **PM**: FA 15, TE 4.75, TR 2100, ST 1, FOV 614.4, MS 240*256, PS 1.0 iso, PB130; **IAT**: 8:37
• **T2w fs FLAIR **2D cor: TIRM; **PM**: FA 150, TE 120, TR 9000, TI 2500, ST 5, SBS 5.5, FOV, MS 288*384, PS 0.599 iso, PB 150; **IAT**: 4:43
• **DWI **(with **ADC**): **SPAIR **epi b-value 0-1000, 2D tra; **PM**: FA 90, TE 98, TR 3700, ST 5, SBS 6, FOV 529, MS 256*256, PS 0, 89844 iso, PB 1000; **IAT**: 0:49

**functional MRI (fMRI) for pain perception: Epi 2d**: epi2d_bold; **PM**: Fa 90, TE 60, TR 2600, ST 5, SBS 6, FOV, MS 384*384 (Start fMRI: 16*16), PS 3.59375, PB 2440; **IAT**: ~15:20 in total

**MRI in module III: Cardiovascular System**

**Cardiac cine-MRI **(PP: HFS, supine), **IAT**: ~25:00 in total
• **Cine SSFP**, 2D: GRE cine with retrospective 2d cardiac triggering; **PM**: FA 80, TE var, TR var, ST 6, SBS 6, FOV 1156/1089, MS 192*192/156, PS 1.771/1.71875 iso, PB 930, IN: 30
• **Phase contrast acquisition 2D; PM**: venc 150, FA 30, TE 2.33var, TR 41.1var, ST 6, FOV, MS 180*192, PS 1.875 iso, PB 555, IN:25
• **Cine-tagging SSFP**, 2D: GRE cine with retrospective 2d cardiac triggering; **PM**: FA 20, TE var, TR var, ST 6, SBS 18, FOV 1073.25, MS 212*256, PS 1.40625 iso, PB 500, IN: 21

**Vascular cine-MRI **(PP: HFS, supine), **IAT**: ~25:00 var in total
• **Carotid artery: FLASH **2D tra: T2*w gradient-spoiled GRE cine with prospective 2d cardiac triggering; **PM**: FA 15, TE ~5.45 var, TR ~34.75 var, ST 6, FOV 289, MS 320 × 320, PS 0.53125 iso, PB 250, IN: 50/RR-cycle; **IAT**: ~5:30 var
• **Femoral artery: FLASH **2D tra: see carotid artery; **PM**: FA 15, TE 5.00 var, TR 26.80 var, ST 6, FOV 768, MS 512 × 384, PS 0.625 iso, PB 250, IN: 50/RR-cycle; **IAT**: ~4:20 var
• **Aortic flow **prox./dist.**: FLASH **2D tra: **PM**: FA 20, TE 2.75 var, TR 11.55 var, ST 5, FOV 768, MS 256*192, PS 1.25 iso, PB 590, IN: 100; **IAT**: ~4:10

**MRI in module IV: Morphometry, Body Composition**

**whole body MRI **(PP: HFP/FFP, prone): **T1w TSE **2D tra; **PM**: FA 180, TE 12, TR 490, ST 10, SBS 20, FOV 1991, MS 256*196, PS 1.9922 iso, PB 120, IN: 90-120 var; **IAT**: appr. 20:00

**MR spectroscopy **(PP: FFS, supine) for **evaluation **of intramyocellular lipids (IMCL): **Single-voxel STEAM**, TE 20, TR 2000, voxel of interest 11 × 11 × 20 mm^3^, 40 acq., **IAT: **appr. 10:00

Tra, transversal (axial); sag, sagittal; **cor**, coronal; Seq., sequence; ADC, apparent diffusion coefficient; bold, blood oxygenation level dependent contrast; DWI, diffusion weighted imaging; epi, echo planar imaging; FLAIR, fluid-attenuated inversion recovery; FLASH, fast low angle shot; fs, fat saturated; GRE, gradient echo seq.; IR, inversion recovery; mpr, MPRAGE (magnetization prepared rapid gradient echo); PDw, proton density weighting; SPAIR, spectral adiabatic inversion recovery; SSFP, steady state free precision; STEAM, stimulated-echo acquisition mode; T1w, T1 contrast; T2w, T2 contrast; TIRM, turbo inversion recovery magnitude; (T)SE, (turbo) spin echo; PM, parameters; parameter abbreviations: FA, flip angle (°); TE, time to echo (ms); TR, repetition time (ms); TI, inversion time (ms); ST, slice thickness (mm), SBS, spacing between slices (mm); FOV, field of view (cm^2^); MS, matrix size (pixel); PS, pixel size (mm); PB, pixel bandwith; IN, image number (n); var, variable dependency; venc, velocity encoded gradient echo imaging (cm/second); IAT, image acquisition time (min:second); PP, patient positioning; HFS, head forward supine; FFS, feet forward supine; HFP, head forward prone; FFP, feet forward prone; cardiac triggering, ECG- gating.

### MRI of feet

**Figure 4:**
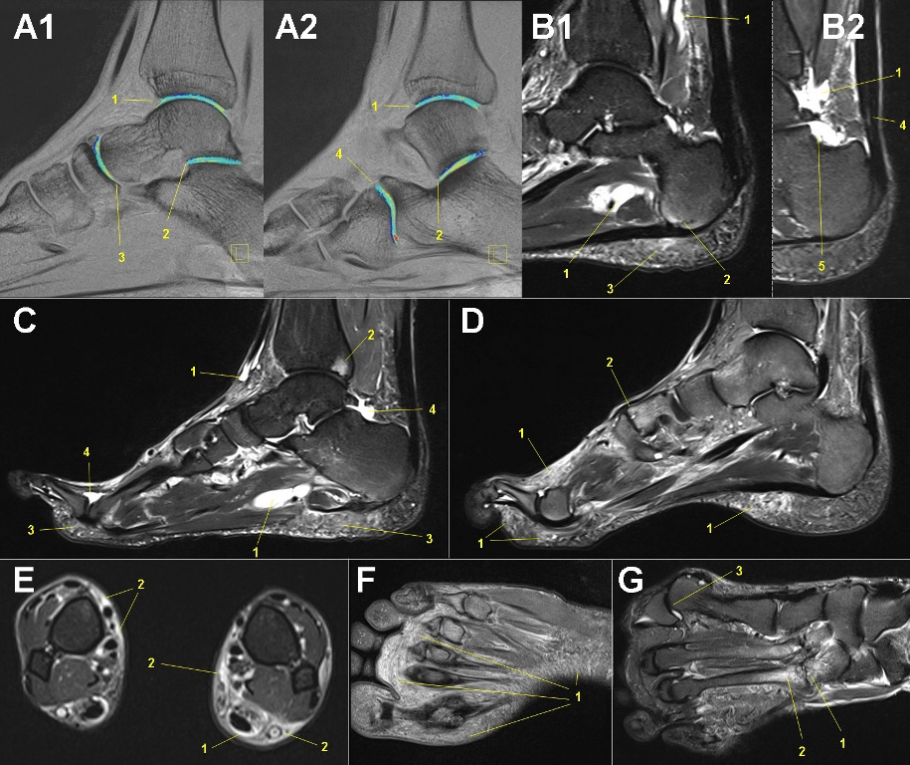
**Mobile MRI of the feet**. **A: **male, 39-years-old, stage 53, 3,669 km (fused colored T2* GRE map sagittal: syngo™ MapIt fusion technique, Siemens Medical solutions, Erlangen, Germany): Colored visualization of focal water concentration in cartilage layers of the ankle joint (1), subtalar joint (2), talonavicular joint (3) and calcneocuboid joint (4). **B: **male, 61-years-old, stage 23, 2,176 km (T2 TIRM sagittal): Multiple running related signs of tissue alteration: Peritendinous fluid accumulation in tendon sheaths (1). Focal bone edema at insertion of plantar fascia (2) with corresponding subcutaneous edema (3), but without plantar fasciitis. Edema in the Achilles tendon (4). Articular effusion in the ankle joint (5). **C: **male, 59-years-old, stage 45, 3,082 km (T2 TIRM sagittal): Peritendinous fluid accumulation in tendon sheaths of foot dorsiflexors (1), focal arthrosis of the ankle joint with subchondral bone edema (2), plantar subcutaneous edema (3), articular effusion in subtalar and metatarsophalangeal joint (4). **D: **male, 54-years-old, stage 32, 2,176 km (T2 TIRM sagittal): Subcutaneous edema (1), arthrosis of midfoot joints (2). **E: **male, 30-years-old, stage 12, 789 km (PDw TSE fs transversal): Peritendinous fluid accumulation in Achilles tendon sheath (1) with wide local subcutaneous edema (2). **F: **female, 68-years old, stage 15, 1,003 km (PDw TSE fs transversal): Extensive plantar subcutaneous edema (1). **G: **male, 47-years-old, stage 52, 3,609 km (PDw TSE fs transversal): Severe arthrosis of midfoot joints (1) with perifocal bone edema (2), hallux valgus (3).

For high resolution investigation of the whole foot a special table fixed boot-like designed 8-channel foot-ankle coil was chosen and a sagittal orientated water sensitive T2w MR sequence (TIRM) configured a wide field of view. If on this sequence any pathology was detected, a transversal oriented focused water sensitive sequence with a more structured T2 sequence (fat saturated proton density weighted (PDw)) was added. For investigation of the joint cartilage a specific T2* mapping MR sequence (syngo™ MapIt FLASH T2*w GRE) in sagittal orientation was used [127-129], allowing quantitative measurement of hydrophilic changes in the cartilage layers of tibiotalar, talocalcaneal, calcaneocuboid, and calcaneonavicular joints. The specification of these MR sequences (Table 2) was done for detection of typical running associated overuse injuries of the feet [52]: subcutaneous edema, Achilles tendonitis [49,50], extensor digitorum tendonitis [48,49]), plantar fasciitis [50], calcaneal apophysitis, arthritis/arthrosis, stress fractures, bone edema, metatarsalgia, Morton's neuroma, and ankle inversion injuries (Figure 4).

### MRI of knees

**Figure 5:**
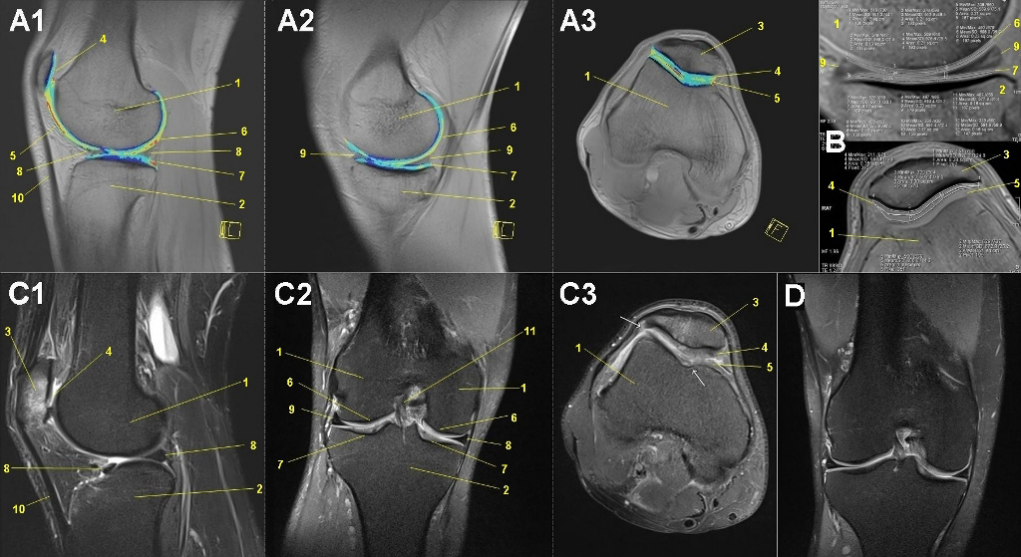
**Mobile MRI of the knees**. Femoral condyle (1), tibial head (2), patella (3), retropetallar cartilage layer (4), ventral femoral cartilage layer (5), dorsal femoral cartilage layer (6), tibial cartilage layer (7), medial meniscus (8), lateral meniscus (9), patellar tendon (10). **A: **female, 45-years-old, stage 44, 3,021 km (fused colored T2* GRE map: syngo™ MapIt fusion technique, A1: medial femorotibial joint sagittal, A2: lateral femorotibila joint sagittal, A3: femoropatellar joint transversal). **B: **male, 25-years-old, before start of TEFR09 Cartilage layer segmentation (T2* GRE map: syngo™ MapIt). **C: **male, 43-years-old, stage 40, 2,738 km (PDw TSE fs, C1: sagittal, C2: coronal, C3: transversal): Severe arthrosis of the patellofemoral joint with retropatellar cartilage defects (4, 5) and wide subchondral bone edema of the patella (3), intrachondral signal alterations of the femoral (6) and tibial (7) cartilage layers. **D: **male, 26-years-old, stage 40, 2,738 km (PDw TSE fs coronal): Nondescript cartilage layers of femorotibial joint.

With a table-fixed 8-channel knee coil all subjects of group 1 had both knees examined with a sagittal TIRM sequence for water detection in knee-related tissues and evaluation of femorotibial joint. A transversal fat saturated PDw sequence was used to assess the femoropatellar joint. As for the hindfoot joints, specific T2* mapping MR sequences in sagittal and transversal orientations were done for quantification of cartilage layers of the femoropatellar and femorotibial joints regarding intrachondral water proportioning [127-129]. The specification of these MR sequences (Table 2) was done to evaluate running-associated overuse injuries in the knees [52]: patella tendonitis ('runner's knee'), arthritis/arthrosis [130], stress fracture, bone edema [64], retropatellar pain syndrome [48-50], chondromalacia patellae, meniscal lesions [50] and patellar tendinitis [50] (Figure 5).

### MRI of hips/pelvis

**Figure 6:**
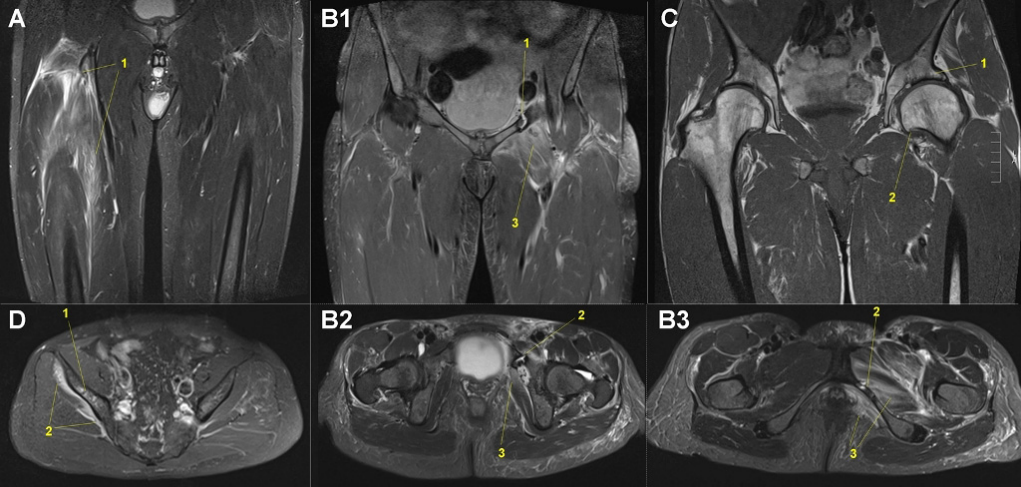
**Mobile MRI of pelvis region and hip joints**. **A: **male, 45-years-old, stage 8, 511 km (PDw TSE fs coronal): Massive edema and (peri-)myositis of right proximal quadriceps muscle (1). **B: **female, 46-years-old, stage 48, 3,290 km (PDw TSE fs, B1: coronal, B2,3: transversal): Stress fracture of left ventral pelvic ring (1: Ramus superior ossis pubis; 2: Ramus inferior ossis pubis) with perifocal soft tissue edema/inflammation (3). **C: **male, 41-years old, stage 11, 739 km (T1 TSE coronal): Arthrosis of the left hip (1: acetabular sclerosis, 2: deformation and osteophyte of the femoral head). **D: **male, 61-years-old, stage 38, 2601 km (PDw TSE fs transversal): Intraosseus edema in the right Ala ossis ileum (1) with massive peri-osseal inflammation of the gluteal muscle origin (2).

One flexible 6-channel body matrix coil was used to obtain an MR overview of the pelvis with one coronal water sensitive sequence (TIRM: Table 2) to detect injuries in this part of the body: hip arthritis/arthrosis [131], sacroiliac injuries [52], stress fractures of the pelvic ring [132-134], muscle overuse injuries and so on. Additional case specific sequences were added as necessary (Figure 6).

### MRI of upper/lower legs

**Figure 7:**
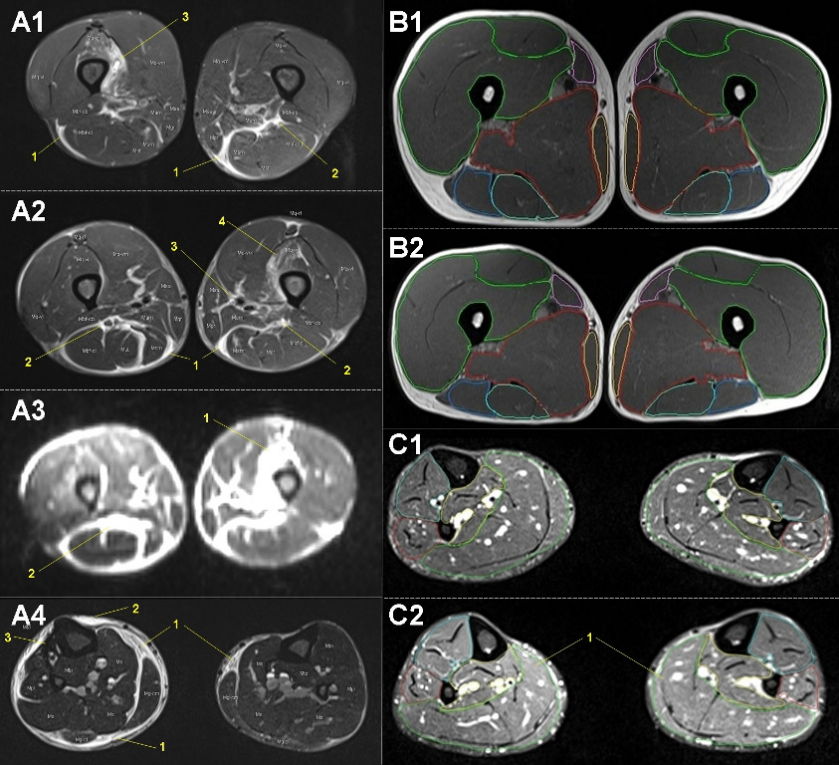
**Mobile MRI of upper and lower legs**. **A: **male, 49-years-old (coronal slices): A1: stage 12, 789 km, upper legs (PDw TSE fs): Subfascial intermuscular fluid, superficial (1), deep peri-neural (2). Partial quadriceps tear (M. vastus intermedius: 3). A2: stage 19, 1,260 km, upper legs (PDw TSE fs): Subfascial intermuscular fluid, superficial (1), deep peri-neural (2) and peri-vascular (3). Partial muscle edema of M. vastus intermedius (4). Specific diffusion weight imaging (A3, same slice as A2) is a sensitive method for free water detection. A4: stage 19, 1,260 km, lower legs (T2 TIRM): Subfascial intermuscular (1) and epifascial subcutaneous edema (2) indicating soft tissue inflammation such as perimyositis and panniculitis (shin splints), respectively. **B: **male, 31-years-old, B1: start, B2: stage 62, 6,358 km (PDw TSE fs transversal): Segmentation of muscle compartments of upper leg for functional muscle volumetry. **C: **male, 53-years-old: C1: start, C2: stage 46, 3,161 km (T2 TIRM transversal): Segmentation of muscle compartments of lower leg for functional muscle volumetry. Muscle edema in calf muscle (1).

With three to four flexible 6-channel body matrix coils total MR examination of upper and lower legs was possible. To get detailed information about soft tissue edema, muscle perfusion and injuries of the legs different sequences were adapted in transversal orientation (T1w for adipose tissue separation and acute bleeding detection, TIRM for high sensitivity in water detection, fat saturated PDw for structural detailed water sensitive imaging, DWI for perfusion analysis of muscles and separation between intra- and extra-cellular water in the muscles: Table 2). With these sequences all of the typical running-associated syndromes could be detected and differential diagnosis done [49,52]: anterior compartment pain/syndrome [48], (medial) tibial stress syndrome [50,135,136], gastrocnemius injuries, peroneal tendonitis, tibialis posterior injury, calcaneal apophysitis, iliotibial band friction syndrome [50], greater trochanteric bursitis, gluteus medius - hamstring - adductor - abductor - quadriceps injuries, such as tendonitis, strains or tears. Muscle volumetry of different compartments of the upper and lower leg muscles is possible for evaluation of changes in muscle volume: Figure 7.

### Cerebral MRI, functional MRI

**Figure 8:**
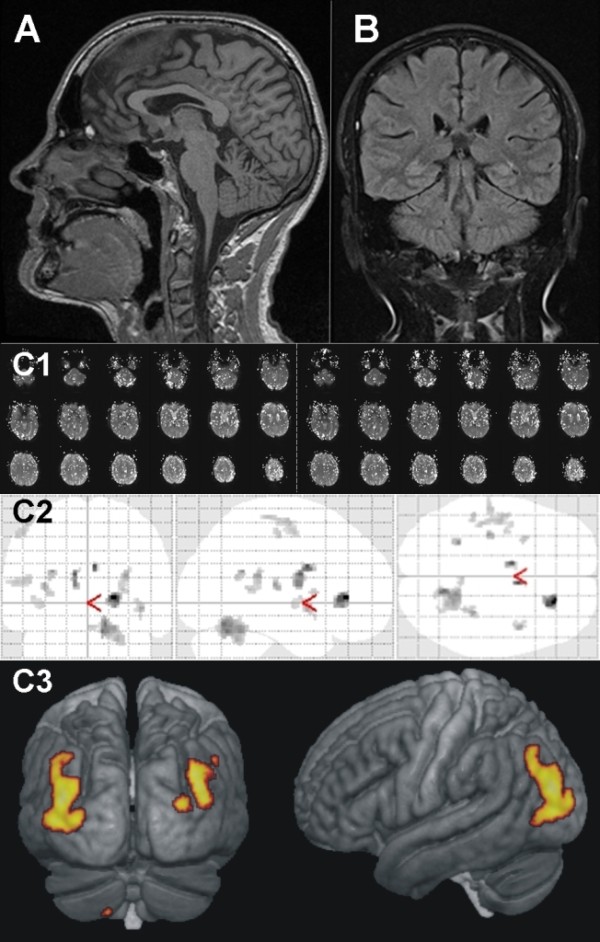
**MRI of the brain**. Male, 52-years-old. **A1: **stage 57, 3,971 km (T1w turbo FLASH 3D sagittal): High resolution isometric three-dimensional sequence (1 mm) allows isovoxel based volumetry (VBM) of the whole brain. **A2**: Three-dimensional view shows dominant areas of volume loss (colored) of grey brain matter occurring during the TEFR09. **B: **stage 36, 2,448 km (T2w FLAIR): Sensitive sequence for detection of brain lesions. In this case, no lesions visible. **C: **20 days before the start. Functional MRI (fMRI) using blood oxygenation level dependent (BOLD) contrast for evaluation of pain perception in ultra runners (C1: without pain stimulus, C2: with pain stimulus). C3: Post-processing analysis using statistical parametric mapping (SPM) shows areas of activation.

As for the muscles in the legs, a MRI guided volumetric analysis of the brain was one focus of the cerebral MRI measurements. Therefore, a T1 weighted high resolution (1 mm) turbo FLASH three-dimensional-sequence was used, making an isovoxel based volumetry (VBM) possible (Figure 8A). For detection of brain lesions and global edema a typical T2-sensitive sequence (FLAIR) in coronal orientation was chosen (Figure 8B). With diffusion weighted imaging (DWI), ischemia detection was possible. For all these MR sequences (Table 2) a table integrated 12-channel head matrix coil with a head restraint system was used. The same coil was used on the stationary scanner for functional MRI (fMRI) using echoplanar imaging (epi) with blood oxygenation level dependent (BOLD) contrast to analyze pain perception in 12 participants of the TEFR09 compared to age-related normal volunteers (Figure 8C).

### Cardiac cine MRI

**Figure 9:**
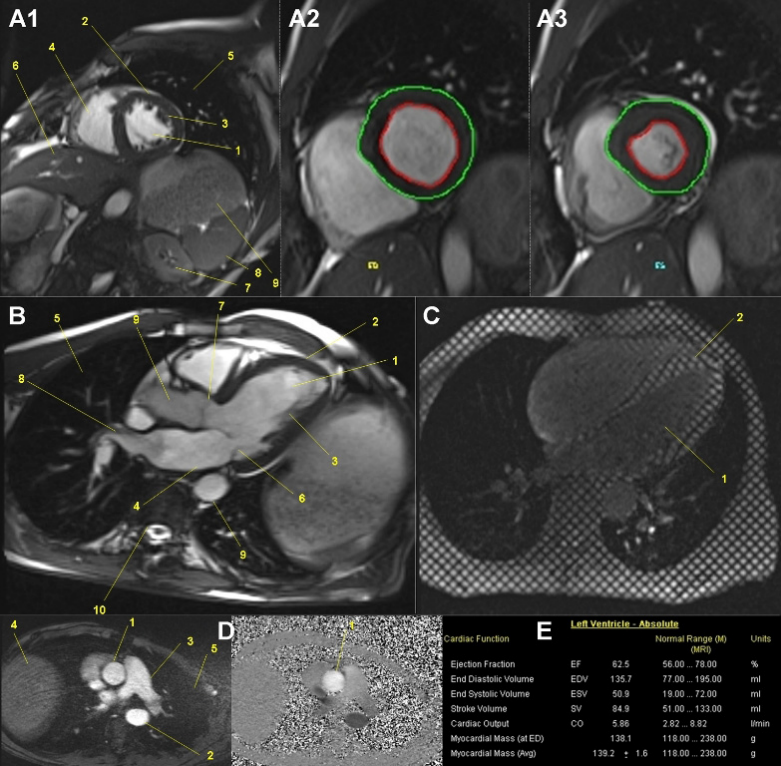
**Mobile cardiac cine-MRI**. **A: **male, 52-years-old, stage 23, 1,569 km (cine SSFP GRE, 2-chamber view): Focus is the left ventricle (1), myocardium (2). papillary muscles (3), right ventricle (4), lung (5), liver (6), left kidney (7), spleen (8), stomach (9). A2, A3: Specific post-processing makes functional volumetry of left ventricle and myocardium possible (green line: epicardium, red line: endocardium). **B: **male, 52-years-old, stage 25, 1,706 km (cine SSFP GRE, 3-chamber view): left ventricle (1), myocardium (2), papillary muscles (3), left atrium (4), lung (5), mitral valve (6), aortic valve (7), pulmonary vein (8), aorta (9), thoracic spine (10). **C: **male, 49-years-old, stage 26, 1,770 km (cine tagging SSFP GRE, four-chamber view): MR tagging of the left ventricle (1) makes quantification of the myocardial (2) motion with its spatial orientation possible. **D: **female, 45-years-old, stage 38, 2,601 km (phase contrast transversal): Ascending aortic (1) flow is measured by specific velocity-encoded (venc) MR imaging. Descending aorta (2), pulmonary artery (3), liver (4), lung (5). **E: **Selection of possible cardiac parameters measurable by cardiac cine-MRI.

For mobile cardiac cine MRI, a flexible six-channel body matrix coil was used. Cine SSFP gradient echo sequences with retrospective cardiac triggering were generated to obtain plane short axis four-, three- and two-chamber (Figure 9B) views of the heart. The mitral and aortic flow (Figure 9D) was measured using phase contrast sequences with 150 cm/second velocity encoded gradient echo imaging (venc). This protocol ensured measurement or secondary evaluation of parameters, such as ejection fraction (%), end diastolic and systolic volume and, therefore, stroke volume (ml), cardiac output (L/minute), myocardial mass (g) (Figure 9E), muscle volume of ventricles (ml) (Figure 9A), and so on. MR tagging using a Cine SSFP gradient echo sequence with retrospective cardiac triggering in plane short axis four- and two-chamber view (Table 2) made quantification of the myocardial motion with its spatial orientation (Figure 9C) possible.

### Vascular cine MRI

**Figure 10:**
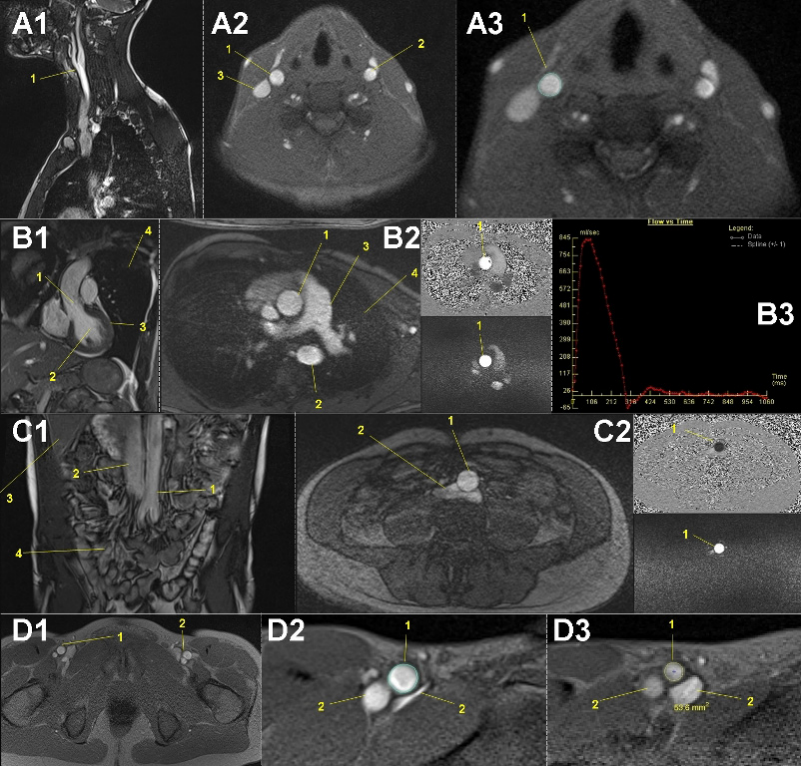
**Mobile vascular cine-MRI: male, 52-years-old, stage 27, 1,838 km**. **A1: **MR localizer sagittal, A2,3: FLASH transversal: Automatic functional measurement of common right carotid artery diameter just below carotid bifurcation (1). Left carotid artery (2), right deep jugular vein (3). **B1: **MR localizer, **B2: **phase contrast transversal: Ascending aortic (1) diameter and flow is measured by specific velocity-encoded (venc) MR imaging. Descending aorta (2), pulmonary artery (3), lung (4). **B3: **graphic depiction of aortic pulsatile flow (ml/second). **C1: **MR localizer coronal, **C2**: phase contrast transversal: Distal descending aortic (1) diameter and flow is measured by specific velocity-encoded (venc) MR imaging just above the aortic bifurcation. Inferior vena cava (2), liver (3), intestines (4). **D1: **FLASH transversal: Functional measurement of superficial right femoral artery diameter just below bifurcation (1), left femoral artery (2). **D2: **Manual diameter measurement (1), **D3: **Automatic diameter measurement (1). Femoral veins (2).

For analysis of changes in the arterial aortic stiffness, measurement of the central pulse wave velocity using MRI is the gold standard [137]. With detection and measurement of the proximal and distal aortic flow and diameter using phase contrast acquisition with venc and prospective two-dimensional cardiac triggering on mobile MRI (Figure 10B, C), this and the central hemodynamic changes (peak and mean shear rate differences) and their influence on the vascular (aortic) diameter [100] during the TEFR09 can be calculated. Additionally, T2 weighted cine FLASH gradient echo sequences with prospective two-dimensional cardiac triggering were generated (Table 2) to measure compliance changes of the vessel wall of the distal common carotid (Figure 10A) and proximal superficial femoral artery (Figure 10D). In total, for vascular MRI three flexible six-channel body matrix coils for aortic and femoral artery measurements, one four-channel phased dual mode neck matrix coil and ECG triggering makes positioning and preparation of the subjects very time consuming.

### Whole body MRI

**Figure 11:**
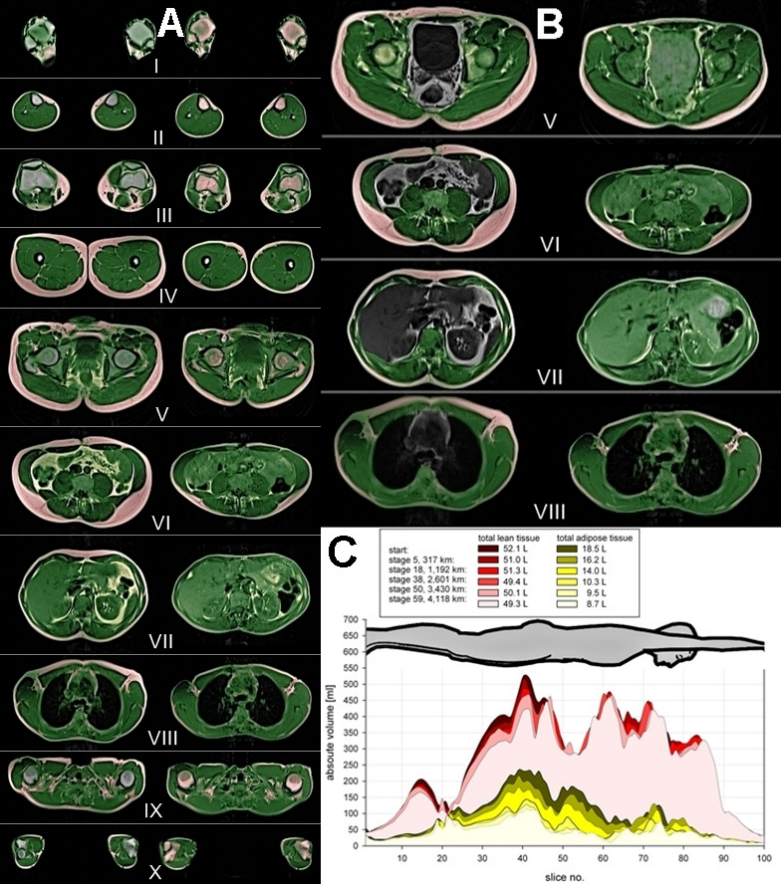
**Semiautomatic tissue separation with mobile whole body MRI of a 32-year-old male finisher of the TEFR09**. **A: **Right row (before start): green: total lean tissue, red: somatic adipose soft tissue, yellow: visceral adipose tissue, blue: adipose bone marrow. Left row (after 4,120 km): green: total lean tissue, red: somatic adipose tissue (= somatic adipose soft tissue + adipose bone marrow), yellow: visceral adipose tissue. (selected slices: I: ankles, II: middle of lower legs, III: knees, IV: middle of upper legs, V: hip/pelvis, VI: umbilical level, VII: upper abdomen, VIII: heart/mediastinum, IX: shoulder girth, X: elbows). **B: **Right row (before start): green: somatic lean tissue, red: somatic adipose tissue, grey: total visceral volume. Left row (after 4,120 km): green: total lean tissue, red: somatic adipose tissue (= somatic adipose soft tissue + adipose bone marrow), yellow: visceral adipose tissue, blue: intraluminal nutrition fat in intestinal tract. (selected slices: V: hip/pelvis, VI: umbilical level, VII: upper abdomen, VIII: heart/mediastinum). **C: **Loss of total lean and total adipose tissue during the TEFR09.

For total body MRI, change of subject positioning from prone head forward to prone feet forward was necessary during a T1 weighted turbo spin echo sequencing using an adapted protocol developed on adipose and diabetic volunteers [138] (Table 2). With topographic tissue segmentation and mapping of the athlete's body using a fuzzy c-means algorithm according to Würslin *et al*. [139] a simple and time-saving strategy for assessment and standardization of the tissue distribution in the entire body was possible. With additional manual adaption due to the non-fasting condition of the subjects changes in different lean and adipose body compartments could be measured during the TEFR09 (Figure 11).

### MR-spectroscopy

**Figure 12:**
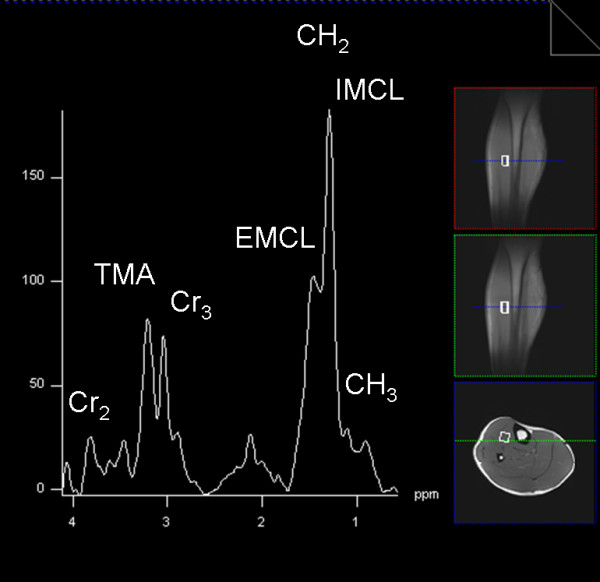
**Mobile MRI H^1^-spectroscopy**.

Proton MR-spectroscopy with a flexible six-channel body matrix coil for measurement of the intramyocellular lipid (IMCL) content of the tibialis anterior and soleus muscle required the stimulated-echo acquisition mode (STEAM) technique (Table 2) and manual shimming of the magnetic field [140], which makes generation of valuable results on a mobile MRI difficult and unpredictable (Figure 12).

### Focused supplementary sequences

**Figure 13:**
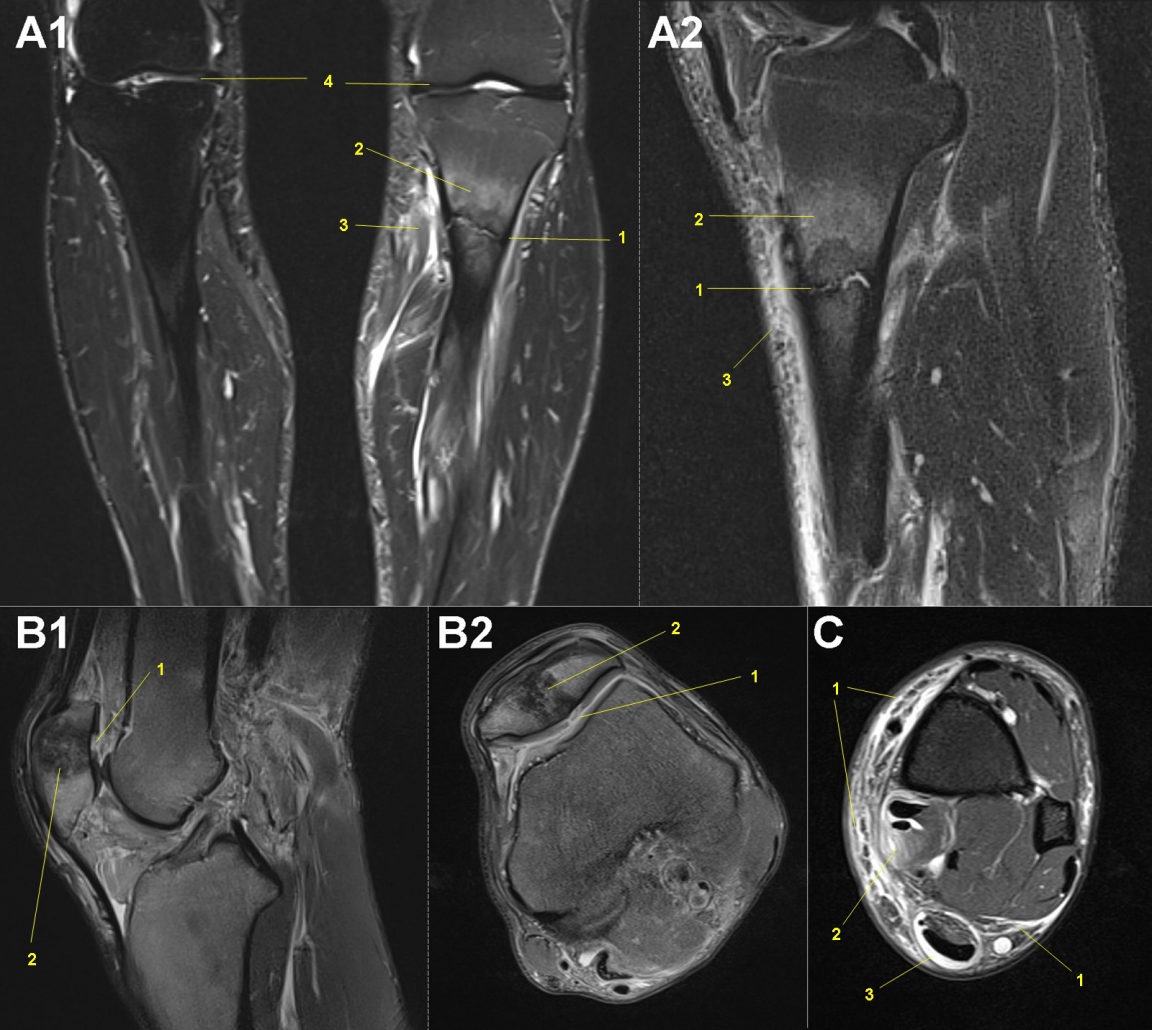
**Supplementary mobile MRI examinations during the TEFR09**. **A: **male, 61-years-old, stage 38, 2,601 km (PDw TSE fs, A1: coronal, A2: sagittal): Stress fracture of the proximal tibia (1) with perifocal bone edema (2) and focal subcutaneous edema (3). **B: **male, 49-years old, stage 52, 3,609 km (PDw TSE fs, B1: sagittal, B2: transversal): Retropatellar chondral ulcer (1) leading to bleeding/hematoma of the patellar bone (2). **C: **male, 41-years-old, stage 13, 857 km (PDw TSE fs transversal): Massive subcutaneous edema (panniculitis: 1) with focal myositis of deep flectors (2) and tenosynovitis of Achilles tendon (3).

In addition to the mentioned study protocol, additional MR examinations were done on subjects and TEFR participants, if acute injuries (for example, stress fractures [52]) and pain syndromes (for example, low back pain [49,52]) occurred and a specific diagnostic finding was necessary to prevent further injuries or complications on the endurance runners (Figure 13).

### Anthropometric and cardiovascular physical measurements

Anthropometric and cardiovascular physical measurements were done on all subjects (Figure 3) every fourth day. Therefore, the 44 subjects were randomly assigned to one of four groups. Body mass was measured with BIA using a Tanita BC-545™ BIA scale (Arlington Heights, IL, USA: to the nearest 0.1 kg). This balance gave additional results about percentage of body fat and lean body mass based on MR validated calculation procedures [141]. The measurements took place in the morning (between 4 a.m. and 5 a.m.) and after the stage (between 3 p.m. and 9 p.m.) together with measurement of blood pressure and body temperature (T) using an infrared ear thermometer (ThermoScan IRT 4020 ™, Braun, Germany: to the nearest 0.2°C. After the stage between 3 p.m. and 9 p.m., the skinfold (SF) thickness of the same subjects was measured using a skinfold caliper (GPM ™, Silber and Hegner, Zurich, Switzerland: to the nearest 2 mm) and their segmental body circumference (CF) was measured using a retractable measuring tape (to the nearest 1 mm). For SF, the mean value was calculated from three consecutive intra-individual measurements at eight regions on the right side of the body according to Ball *et al*. [142]: chest, midaxillary (vertical), triceps, subscapular, abdominal (vertical), suprailiac (at anterior axillary), thigh, and calf. For CF, mean value was calculated from three consecutive intra-individual measurements at six regions on the right side of the body according to Lee *et al*. [143]: upper arm (largest part of the limb), waist, hip, thigh (10 cm/20 cm above upper patella pole), and calf (largest part of the limb). To avoid inter-observer error all the anthropometric measurements were done by the same, specifically trained investigator. Every 800 km a short term ECG was planned on every subject.

### Lab samples

Midstream urine samples were taken from all subjects twice each day. Before breakfast in the morning between 4:00 a.m. and 5:00 a.m. and after each stage in the evening after dinner between 7:00 p.m. and 9:00 p.m. Blood samples were taken every 1,000 km from the cubital vein after stage. The samples were immediately centrifuged and frozen (below -20°C) and put on -80°C after the race.

### Post-race/follow-up

On the day they dropped out, non-finishers (NF) had a last complete measurement of all specific MRI protocols and physical examinations (BIA, SF, CF) and provided blood- and urine-samples. Nearly eight months after the TEFR09, 15 of the 44 subjects (all of them finishers of the TEFR09) had a follow up examination at UHU on the same topics involved during the field studies: specific MRI examinations, anthropometric measurements, ECG and blood and urine samples.

### Statistical analysis

For statistical analysis the software 'SPSS 12.OG for Windows, Version 12.0.1' was used. Data are presented as mean (SD, range) and median (IQR) as appropriate. The coefficient of variation (CV (%) = 100*SD/mean) was calculated only for measured absolute data on performance. The stage severity index (SSI) is an indirect parameter calculated from the mean stage velocity of all runners without a severe handicap v;¯ _STAGE* _in relation to the total mean velocity of the whole race v;¯ _TEFR*_. Therefore, the SSI represents the relative burden of each stage, which is dependent on the mentioned multiple external factors that changed daily. It reflects the sum of daily weather and route conditions:

$$\text{SSI}=\;{\text{v}}_{\text{STAGE}}\frac{{\bar{\text{v}}}_{\text{STAGE*}}\;}{{\bar{\text{v}}}_{\text{TEFR*}}}$$

*: values are only integrated in calculation, if the stage performance of the specific runner is more than 87% of his mean race performance

## Results

### Race conditions

**Figure 14:**
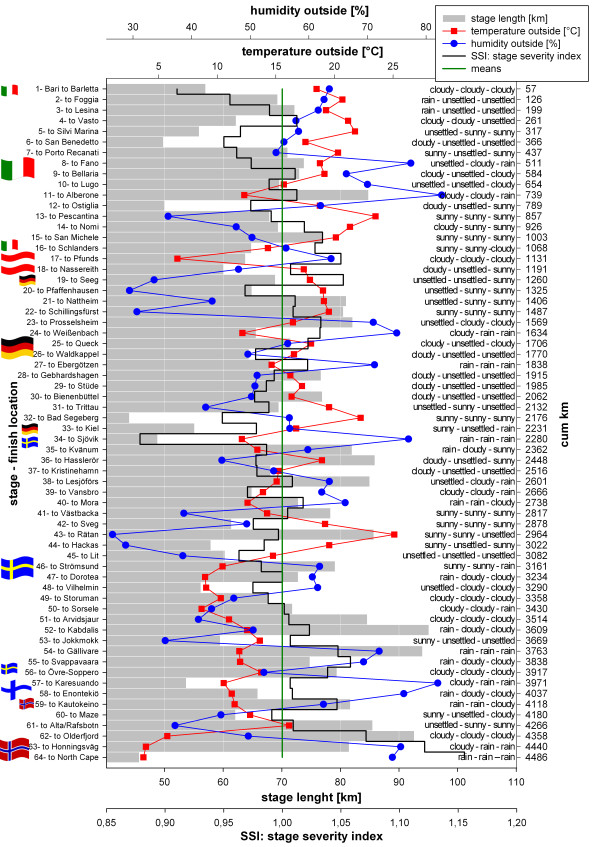
**Daily profile of weather and stage conditions during the entire TEFR09**.

The mean stage length was 70.1 km (SD 11.8 km, range 44 to 95.1 km) and influenced the SSI positively (Figure 14). Temperature and humidity were also factors influencing the SSI and showed a mean (mean of three daily measures at 6:00 a.m., 10:00 a.m. and 2:00 p.m.) of 15.2°C (SD 4.7°C, range 3.7 to 25.1°C) and 55.6% (SD 14.3, range 26.5% to 82.7%), respectively. Altitude differences were not measured. The longest stages occurred in the last third of the race and the coldest, wettest and most humid and, therefore, most severe stages, were at end of the TEFR09 which pushed the runners to their limits (Figure 14).

### Changes of study plan due to hazards in TEFR

**Figure 15:**
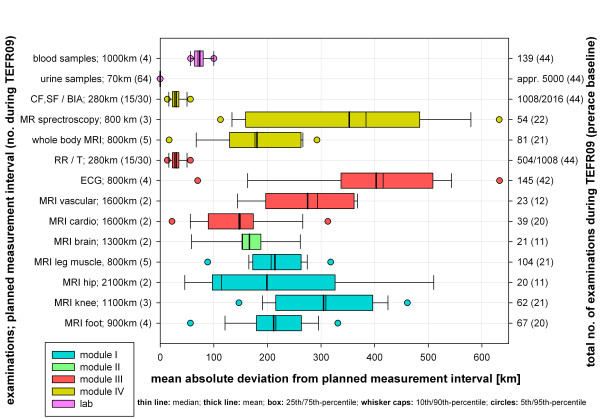
**Deviation of measurements from projected intervals [km] during TEFR09**.

For every research topic, distance intervals of measurement (MI) throughout the TEFR09 were defined. The discrepancies between these planned and the realized MI can be shown as mean absolute deviations (Figure 15). For MRI, data showed mean deviations between 100 and 300 km. For MR spectroscopy it raised up to 400 km, because this special MR technique was highly dependent on the locations with their local magnetic field disturbances (such as, traffic and so on). However, reasons for the deviations were multifold.

Study staff had to deal with many influencing factors, which made daily adaptation of the research plan necessary: acute or chronic illness of study staff, bad weather conditions (Figure 14) which sometimes influenced operability of the mobile MRI, accidents and technical problems (Table 3) and local situations at stage destination which sometimes made a nearby commissioning of the mobile MRI difficult. However, the strongest influence forcing the staff to change and adapt the daily research work program was the athlete with more or less daily changes in mental and physical conditions and necessities: pain, fatigue, fears, doubts, illness, nutrition time schedules and specific behavior and rituals regarding regeneration from this immense physical and psychological stress. Therefore, it was not always possible to ensure the exact time of pre- and post-race measurements. Despite these uncertainties, 95% of the measurement protocol could be followed.

**Table 3:** Relevant accidents and damage to MRI and vehicles during the TEFR project

**No**.	stage	location (**Figure 1**)	event (**Figure 3**)	MR down time
**1**	0	Bari, Southern Italy	Defect of MRI table.	24 hours
**2**	12	Lugo to Alberone, Northern Italy	Truck collision on bridge over the river Po.	-
**3**	33	Bad Segeberg, Nothern Germany	Roof damage on MRI trailer.	-
**4**	36	Göteborg to Sjövik, Southern Sweden	Total system breakdown, damage of one compressor.	16 hours
**5**	38	Kristinehamn, Central Sweden	Truck sunken in football sand-field.	5 hours
**6**	45	Hackas, Central Sweden	Severe ankle fracture of MRI assistant.	16 hours
**7**	56	Svapparaava, Northern Sweden	Total rupture of tractor to trailer cables.	2 hours
**8**	after race	nearby Gällivare, Northern Sweden	Reindeer collision on the way back from North Cape: total damage of material van.	-

**Figure 16:**
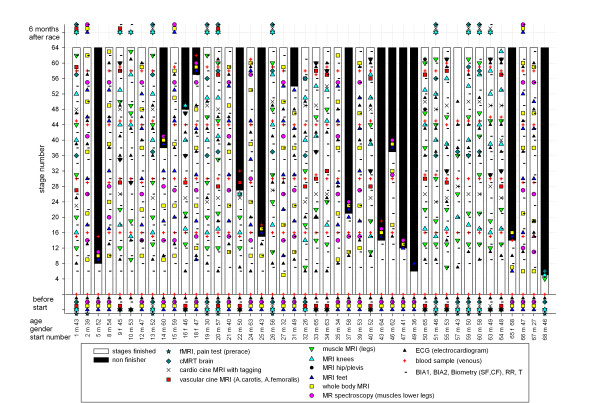
**Realized clinical and MRI-measurements on all subjects during TEFR09**.

Figure 16 shows all performed examinations and measurements done before, during and six months after the TEFR09. The overall work load includes: 741 MRI protocols with 2,637 MRI sequences (more than 200,000 picture data), 5,600 urine samples, 1,018 BIA-, 539 SF- and CF-measurements, 250 blood samples and 205 ECGs.

### Baseline characteristics and performances of subjects

The baseline characteristics of the study groups are summarized in Table 3. Age and gender did not differ between the two MR groups. None of the 44 subjects showed different AROM of the hip and knee or any functional or anatomical mal-alignment of the legs compared to normal values or between the MR groups. Only active hip extension shows a tendency to be better in endurance runners (Table 4).

**Table 4:** Baseline characteristics of the TEFR study population

	all subjects	MR group 1	MR group 2
	number (%)	number (%)	number (%)
total	44	22 (50.0)	22 (50)
men	40 (90.9)	20 (90.9)	20 (90.9)
women	4 (9.1)	2 (9.1)	2 (9.1)
Finisher (F)	30 (68.2)	19 (86.4)	11 (50.0)
Non-finisher (NF)	14 (31.8)	3 (13.6)	11 (50.0)
	mean/median (SD)	mean/median (SD)	mean/median (SD)
age (years)	49.7 (10.5)	50.3 (9.6)	49.1 (11.5)
prerace history:			
years of regular endurance running	17.9 (7.5)	19.1 (7.5)	17.1 (7.4)
finished marathons	91.7 (168.6)	62.0 (93.4)	121.47 (218.8)
finished ultra-marathons	85.4 (63.6)	81.1 (59.0)	89.8 (69.0)
finished multistage ultra-marathons	5.7 (3.6)	5.1 (4.1)	6.3 (2.9)
anthropometry:			
height (cm)	175 (8)	175 (6)	174 (9)
BMI (kg/m^2^)	23.1 (2.2)	22.8 (1.8)	23.4 (2.6)
body fat percentage, BIA (%)	11.2 (4.3)	11.0 (4.1)	11.4 (4.5)
body fat percentage, calculated^a ^(%)	16.6 (4.2)	15.5 (3.2)	16.6 (5.0)
body fat percentage, MRI (%)	-	-	22.7 (6.0)
muscle percentage, calculated^b ^(%)	49.8 (5.1)	49.7 (4.7)	50.0 (5.7)
somatic lean tissue, MRI (%)	-	-	65.0 (5.3)
active range of hip motion (°)			
flexion, 121 (26)^c^	123 (27)	122 (26)	124 (27)
extension, 19 (16)^c^	24 (17)	25 (17)	21 (16)
abduction, 42 (22)^c^	43 (23)	43 (22)	42 (24)
internal rotation, 31 (16)^c^	31 (16)	30 (16)	32 (16)
external rotation, 32 (18)^c^	34 (19)	33 (18)	34 (19)
active range of knee motion (°):			
flexion, 132 (20)^c^	134 (19)	135 (20)	133 (19)
**l**ower limb alignment:	-		-
LL difference [mm], 6 (95^th^: 11)^d^		2 (3.3), 95^th^: 9)	
FTR, 1.26 (0.05)^d^		1.17 (0.04)	
FTA [°], m: 178 (174-182)^e^		178 (175-182)	
w: 181 (177-185)^e^			
FTA difference		1 (0.8)	
MAD (mm), 10 (4-16)^e^		10 (4-17)	

**^a^**calculated by the updated DC (DEXA criterion)-equation according to Ball *et al*. [142], inputs: age, 7SF (chest, midaxillary, triceps, subscapular, abdomen, suprailiac, thigh); ^b^**c**alculated estimation of skeletal muscle mass according to Lee *et al*. [143], inputs: gender, age, race, height, 3 SF and CF (upper arm, thigh, calf); ^c^normal AROM-values of hip and knee joints (goniometric data) according to the 1^st ^National Health and Nutrition Examination Survey (NHANES I) [144]; ^d^normal values of side differences in the lower limb [119], generated with computed tomography gold standard method [117]. FTR: femoro-tibial length-ratio. Leg length (LL) differences of > 14 mm (3SD) are seen as pathological [122,119,118]; ^e^normal values of femorotibial angle (FTA) and mean axis distance (MAD) [116,120,121]

**Figure 17:**
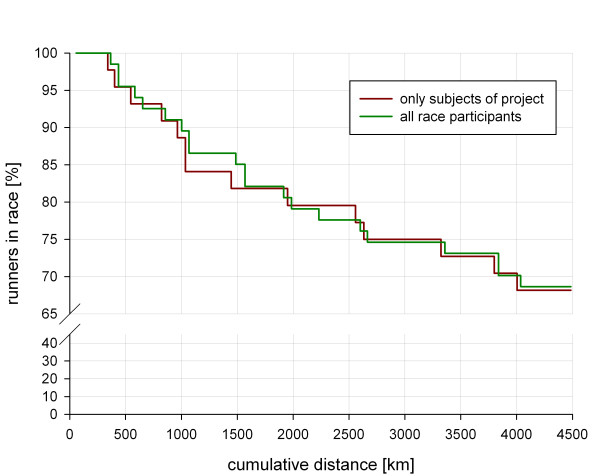
**Drop-out rate of TEFR09**.

Regarding the ratio of F and NF there is no relevant difference between subject group and the whole starter group (Table 5, Figure 17), but randomly between the MR groups (Table 4). Reasons for dropping out of the race were multifold (Table 5, Figure 17). The main reasons for premature exiting the race were overuse syndromes of the soft tissues of the leg, resulting in (peri-)myotendinous inflammations in the lower and upper legs (71.4%). Two subjects suffered a stress fracture in the third part of the race, one high tibia fracture (male, 60 years old) and one ventral pelvic fracture (female, 46 years old). Due to the unspecific pain and the high pain level, they ran with these fractures for about 200 km to 240 km before they gave up. There was one case of a rapidly ascending soft tissue abscess of the upper extremity due to an initially minor finger lesion (male, 39 years old) indicating the immense burden of the ultra-endurance performance to the runners and their immunological system.

**Table 5:** Reason for not-finishing the TEFR09

affected region	pathology	subjects **(number = 14**, 31.8%)	all (number = 21, 31.3%)
Soft tissues of legs:		10 (71.4%)	14 (66.7%)
lower legs:	shin splint: myofasciitis, tenditis	5 (35.7%)	7 (33.3%)
	Achillodynia	-	1 (4.8%)
upper legs:	myo-tendino-fasciitis, perineuritis	5 (35.7%)	6 (28.6%)
Bone/joint of lower body:	stress fractures: tibia, pelvis	2 (14.3%)	2 (9.5%)
	bunion (arthritis)	1 (7.1%)	1 (4.8%)
Upper extremities:	Phlegmon of the hand	1 (7.1%)	1 (4.8%)
Gastrointestinal (GIT):	upper GIT-bleeding (NSAID)	-	1 (4.8%)
	GIT infection	-	1 (4.8%)
Mental problems:	Intolerance of crowded small halls at night	-	1 (4.8%)

GIT, gastrointestinal tract; NSAID, non-steroidal anti-inflammatory drug.

### Performances

**Figure 18:**
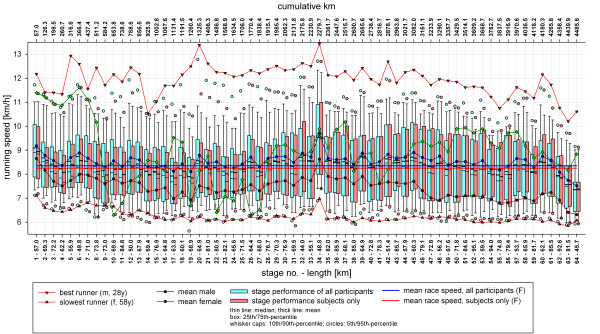
**TEFR09 performances**.

Regarding all participants, the mean speed per stage was 8.35 km/hour (SD = 0.32; CV = 3.8%) and the mean total race speed of all finishers was 8.25 km/hour (SD = 1.4, CV = 17.1%). Finishers invested 552 hours (SD = 91, CV = 16.5%) for the 4,486 km in total. There was a wide range of performance difference between the best and slowest runner throughout the whole race, independent of the stage severity (Figure 18). The best runner (male, 28 years old) performed the race with a mean speed of 11.9 km/hour (total running time: 378 hours), nearly twice as fast as the slowest runner (female, 58 years old), with a mean speed of 6.2 km/hour (total running time: 723 hours. In the subject group, mean speed per stage was 8.28 km/hour (SD = 0.33; CV = 3.9%) and the mean total race speed of the subject finishers was 8.25 km/hour (SD = 1.3, CV = 15.3%), ranging from 11.1 km/hour (male, 26 years old, total running time: 407 hours, second rank) to 6.5 km/hour (male, 63 years old, total running time: 696.4 hours, 45^th ^rank). Subjects mean stage speed was on average 8.32 km/hour (SD = 0.33, CV = 3.9%). Figure 18 shows mean performances in total and per stage.

## Discussion

### History

Looking at transcontinental footrace history, the finisher rate ranges from 28% to 73% (Table 1). The Bunion Derbies of the early twentieth century showed the lowest rate due to lower standards regarding sports equipment, nutrition features, endurance-associated behavioral knowledge and the level of organization. These two Derbies had 150 starters in mean, much more than nowadays, indicating a high rate of rookies with little or no experience in ultra running. In the TEFR09, 31% of the participants (32% of the subjects) did not reach the finish line (Table 5, Figure 17). This is 18% more finishers than at the TEFR03. This could be attributed to the longer distance in 2003 (+540 km) from Lisbon to Moscow, implying a mean difference of 8.3 km per stage between the TEFR09 and the TEFR03. Apart from the TEFR03, running distances of modern transcontinental footraces (1992 to 2009) were approximately equally long and the finishing rate of 68% in the TEFR09 lies in the upper range of the published data (Table 1). Looking at the rate of participation, being more than 200% higher than the mean starter rate of all modern transcontinental footraces, the comparably high finishing rate indicates a professional organization and preparation of both the runners and organizers of the TEFR09.

### Performance

Due to the diverse ways and possibilities to exercise long distance running (that is, area, length, altitude, distance, weather, indoor/outdoor, on/off-road, looped course, combinations with other disciplines and so on), it is extremely difficult to compare the performances of ultra athletes in the literature [107,108,145]. Regarding the present literature, an abundant variety of physiological, anthropometrical, pre-race and training variables seem to influence running performance and associated injuries, depending upon the length and duration of the races [146-150]. In MSUM, such as the TEFR09, the daily changing environmental conditions have a direct influence on stage performances. In the last days of the TEFR09 weather conditions became more and more difficult towards the destination, North Cape, leading to a marked decrease of mean running speed (Figure 18). Only their indomitable will not to drop out at the end of race after more than 4,000 km of running, kept many emaciated participants in the race.

### Drop out and injuries

Due to the likely multifactorial nature of running injuries, very few firm conclusions can be made based on the existing studies. In general, there are intrinsic factors such as individual biomechanical abnormalities (that is, mal-alignments, muscle imbalance, stiffness, weakness, instability) or extrinsic (mostly avoidable) factors such as poor running technique, improper equipment and improper changes in training extent and mode or duration and frequency of the race burden contributing to overuse injuries [151]. The one year prevalence of running injuries is 55% in male marathon runners; limb overuse injuries are the most common [152]. In UM these entities become much more important. The most common injuries for runners are multiply cited in the literature: anterior knee pain (for example, patella-femoral syndrome), iliotibial band friction syndrome, tibial stress syndrome (shin splint/injuries), plantar fasciitis, Achilles tendonitis and meniscal injuries of the knee [152-156].

Approximately two thirds of NF dropped out of the race in the first half of the TEFR09 (Figure 17). As our results show, the reasons for premature resignation of subjects were different. Conforming to the literature [20,22], in more than two thirds of the cases, overuse injuries of the lower and upper limb were the most common reasons (Table 5). However, these soft tissue overuse injuries occurred not only in less experienced ultra runners, but also in runners who had already successfully finished transcontinental races such as the TEFR03 or the 'Run Across America'. There were only a few subjects and runners without any overuse problems of the limbs in these 64 days. However, not every soft tissue overuse inflammation leads to the cessation of running. Most runners were able to 'overrun' these specific problems. They reduced running speed in adaptation to their problems, used topical application of anti-inflammatory medication and some of them took non-steroidal anti-inflammatory drugs for a few days. With adequate behavior many, but not all, athletes recovered and were able to continue the race. Presumably some athletes could handle more pain than others [20]. An example is one extreme runner, an experienced 49-year-old male subject, who had multiple severe overuse-induced soft tissue inflammations with local muscle fiber rupture forcing him to frequently slow down his speed (Figure 7A). He also showed signs of exertional compartment syndrome, but did finish the TEFR09. His ordeal at the TEFR09 is reflected by the red line in Figure 18. Contrary to other reports, Achilles tendonitis or lower limb joint problems were not a reason for subjects to stop the TEFR09. Further results of module II research topics such as specific personality, temperament, character and pain perception will be presented soon.

### Statement of principal findings

The relevance of results in field studies is determined by the appropriateness of the research questions and hypotheses, by the practicability of methods and measurements and the consistency of their specific implementation and by the correct interpretation of results. Due to the manifold open questions and unproven hypotheses in endurance running, the unique opportunity of doing real time observations of changes in the body of athletes while running at the upper limit in a MSUM was demanding.

The TEFR project was designed to explore inter-individual variability in adaption to the tremendous persisting physical endurance running load on the different organic and functional systems of the body with regard to the lack of breaks and time for regeneration.

All technical equipment was tested by the specific manufacturers on reliability and validity under normal clinical conditions and usage. But daily dismantling, transport and setting up of the mobile MRI hardware sets extraordinary demands which were initially not totally verifiable and calculable. Despite some technical problems and temporal defects (Table 3), our arrival at the North Cape demonstrated the feasibility of accompanying a large group of endurance runners (67) with a mobile MRI and all its necessary equipment ensuring permanent operability during the 64 stage ultra marathon.

Throughout the whole TEFR09 our time schedules for examinations adapted to the daily changing local circumstances and the athletes mental state and problems. To avoid additional stress for the subjects, they could not and were not forced to follow the study protocol strictly. However, the efficiency of this strategy was reflected in the high rate of compliance (98%) until the end of the TEFR09. Only one subject who finished the race left the study at stage 36 (km 2,448) due to personal and, explicitly, not study related problems. Consequently, the completion rate of planned examinations over the whole running distance of 4,486 km was only limited by the drop-out rate of the subjects from the TEFR09 (Table 4, Figure 16). In particular, specific implementation of stationary validated MRI protocols on the mobile MRI on the truck trailer by a team of MRI experts and training of the research staff on the mobile MRI before the start ensures practical experience with the experimental protocols under field conditions and makes modification of them possible where necessary.

### Strengths of the study

The strengths of the TEFR project are the unique chance to do a field study with the large number of 44 subjects, the realization of tests and measurements with the modern technical gold-standard equipment MRI in a daily changing and increasingly harsh and inhospitable environment (Figure 14), the complete baseline control data and the high rate of test completion. Large subject numbers provide the statistical power to discriminate between and, identify associations with, different patterns of adaptation as well as to detect differences in response between subgroups. Matched subject race profiles and baseline measurements before the start of the TEFR09 control for variability of exposure to ascending running distance and, thereby, permit valid inter-individual comparison of responses to this burden (with subjects as their own controls), maximizing the signal (true physiological differences) to noise (variations in exposure) ratio.

The avoidance of invasive or interventional tests on the subjects' mechanisms during the TEFR09 and the descriptive nature of the data may be considered a weakness of this study. However, the variety of outputs from different measurement techniques (for example, functional and cine MRI, physical anthropometrical measurements and laboratory data including proteomics, plasma and urine biomarkers) allows observation of consistent patterns of response that may be strongly suggestive of particular mechanisms.

In module I, for example, measured data of T2*-mapping of joint cartilage (Figure 4 A and 4.2A/B) will allow conclusions on the influence of long distance running on the proteoglycans in the cartilage matrix based on the current experimental experiences [43-45,157]. As in most other mobile MR associated examinations of other modules, additional laboratory analyses using specific parameters on collected blood and urine samples (for example, cartilage oligomeric matrix protein (COMP) [158-165] for joint cartilage research) will give further information for interpretation and verification of image related results.

Another example is the vascular cine MRI studies of module III. In humans, the relationships of blood flow changes to structure, function, and shear rate of conducting arteries have not been thoroughly examined. Therefore, the purpose of the vascular cine MRI study in module III (Figure 10) was to investigate these parameters of the elastic-type, common carotid artery (CCA) and the muscular-type, common femoral artery (CFA) in long-term running, assuming that the impact of activity-induced blood flow changes on conduit arteries, if any, should be seen in these highly endurance-trained athletes. These investigations using the gold standard method, MRI [137], enable further analyses on the current status of insights on the question of structural and functional vascular adaption and associated exercise-induced blood flow changes on endurance training based on sonographic B-mode measurements [100].

The manifold investigations of Knechtle *et al*. on ultra endurance athletes [15-22,113,145] focused on the question which anthropometric parameters of ultra athletes are predictors of ultra endurance performance. These authors postulated some direct connections between specific physical anthropometric markers and ultra endurance performance [16,19,166]. Examinations of module I and module IV (morphometry, body composition) of the TEFR project with its possibility of precise and differentiated morphometric analysis (for example, segmental and functional muscle volumetry) may be able to verify common experiences and to detect relationships between anthropometry and morphometry of endurance athletes and performance in MSUM.

All tissue systems - subcutaneous and visceral adipose tissues, muscles, ligaments, fascia, tendons, bones and cartilage - were studied with special quantitative and qualitative MR techniques. This should help explain how the different tissues react to the severe stress that continued for days and weeks without any pauses for regeneration or even resting phases as two marathon distances had to be completed every day.

Individual performance and ability to deal with injuries and overuse symptoms with regard to decision making for stopping MSUM is a complex psychosomatic process and more or less modulated by character traits. Strong changes of endocrine and metabolic status during marathon runs are described [78,79]. Hormonal changes can influence pain sensation and show an influence on specific brain functions [167]. Knowing this, investigations detecting reasons for dropping out of the race (14 subjects) can focus not only on MR image analysis, but must also include specific laboratory analysis and psychometric tests as done or planned in the TEFR project. Serotonin, tryptophan and endorphin are described for use as stress markers in UM [3]. The relation of branched-chain to aromatic amino acids as a model (amino acid dysbalance hypothesis) to explain running-associated fatigue is described [80]. The reduction of the pain sensation is known for cortisol [167]. Considering all these particular mechanisms influencing performance and decision making in ultra athletes, the important dimension of laboratory analysis possibilities, in addition to MR data analysis, becomes obvious for the different parts of the TEFR project.

Overall, the possibility of cross-validations between physical, MR-graphic, -functional and laboratory follow-up data on multiple organic systems during a nearly ten-week ultra run is a unique strength of this study.

### Weakness of the study

The main weakness of this study is the lack of a control group of non endurance experienced subjects. However, this is not a feasible option in field studies under race conditions including such an immense amount of physical and mental load. In order to explore the influence of pre-race running experience, we will undertake subgroup analyses investigating the influence of individual pre-race performances on our findings. In the pre-race pain study (project module II, MR group 2), we recruited a parallel age-related control group that was tested over the same pain scale and functional MRI protocol as the MSUM exposed subjects. This sub-group is, therefore, not confounded by self-selection due to prior endurance tolerance.

As the first attempt in MR research, we tried to perform H^1^-MR-spectroscopy for measurement of IMCL [140,168,169] with a mobile MRI on a truck trailer. MR spectroscopy needs a stable magnetic field and, therefore, a still and static environment around the scanner. Due to the daily changing position of the mobile MRI, the possibility and feasibility of manual shimming was not predictable. This is the only measurement with uncertain validity due to changing environmental conditions in the TEFR project.

Environmental factors, such as ambient weather conditions (Figure 14), subject de- or hyperhydration and concurrent illnesses may also have confounded results. However, indoor temperature (18.7°C, SD 3.0°C, range 11.7 to 28.5°C) and temperature in the MR trailer (20.5°C, SD 0.8°C, range 18.5 to 21.8°C) was much less variable than outdoor temperature (15.2°C, SD 4.7°C, range 3.7 to 25.1°C). All subjects were encouraged to maintain adequate

hydration (guided by the production of good quantities of pale urine). There was only one Japanese subject identified with a severe illness during the race, suffering from a severe cough which persisted from stage 12 till stage 32 (day of drop out).

Another weakness of the study was that there was only rough documentation of nutrition. Nutrition depended on food availability at the TEFR stages and was provided by the TEFR organization. The use of doping substances was forbidden by the terms of participation but not controlled. Runners did not agree to close measurement and documentation of food and caloric intake, because this would have meant too much disturbance of their daily running routine and compromised compliance due to additional stress conducted by the research work. Despite initial concerns, mobile MRI examinations did not result in additional stress for the athletes. On the contrary, most of them enjoyed relaxing in the MR scanner, having no other noises and people around them while listening to their favorite music via headphones.

### Strengths and weakness in relation to other studies

In comparison to previous field, laboratory and radiological, especially stationary MR studies focusing on long distance running and its effects on the human body, our study is unique in several aspects: ultra-long distance running without any day of rest, cohort size of subjects and use of a mobile MRI throughout the whole race. This is the first MR-based follow-up ultra marathon field study that ensures unique data based on repeated measurements on ascending distance burden.

We explored the possibility of conducting this study with a stationary MRI in a fixed local setting. However, this is not realizable with a large cohort size, because not many ultra endurance athletes took the challenge to run ultra long distances in circles in local regions or stadiums day by day. For example, at the Sri Chinmoy Self-Transcendence 3,100 Mile Race over 5,649 laps of one extended city block in Jamaica, Queens, New York (http://www.3100.srichinmoyraces.org) only 10 to 14 participants started regularly. If a study like this is planned, it has to be adapted to the race circumstances and not the race conditions to the study. Only exceptional runners would be willing to take such a burden under laboratory conditions. It is the experience of the distance and the environment that motivates these athletes to run thousands of kilometers. In addition, such an approach might have incurred significant additional costs; our subjects were entirely self-funded, whereas volunteers in chamber studies often expect remuneration.

### Unanswered questions and future research

Further research arising from this study will follow two themes. First, studies in patients to explore the validity of our model by applying the findings of this study to pathophysiological problems in a clinical setting. Second, collecting additional healthy volunteer data from subjects exposed to an ultra endurance burden (ultra marathon, ultra triathlon, ultra cycling and) in further field studies and chamber studies.

Whether it is possible to initiate future projects using this model of a mobile MRI field study is critical. First, this was a unique cohort size in transcontinental ultra running and it would be difficult to find a size like this again: the latest Run Across America (Table 1) had only 14 participants. Second, in addition to sufficient funding a bit of luck is necessary to finish a field study successfully when using a sensitive and high-maintenance technical piece of equipment such as a mobile MRI. Future studies might answer additional questions by using alternative or additional measurement techniques or undertaking novel intervention trials.

## Conclusions

The TEFR project was both a challenge and risk together. It demonstrates the feasibility and safety of conducting a large ultra endurance cohort study with a mobile MRI under 'natural' conditions over 64 stages and daily changing environment on the way across all of Europe. Thanks to the possibility offered by a modern mobile MR-imager diverse research topics from different fields of medicine could be implemented in the measurement protocol to study human adaption to an ultra endurance burden. Systematic measurements of a large set of variables were achieved with high-fidelity in 44 subjects and up to 4,500 kilometers distance running. The resulting dataset is a unique resource for the study of regeneration and adaption in relation to a high impact ultra endurance running burden which may improve specific or general scientific understanding of responses to critical illness at the limits of stress and strain of the human body.

## Competing interests

The authors declare that they have no competing interests.

## Authors' contributions

US contributed to the conception and design of the study, to the funding, to the acquisition of data, the analysis of data, the interpretation of data and drafted the manuscript. AST contributed to the conception and design of the study, to the acquisition of data, and the analysis and interpretation of data. BK contributed to the design of the study and the interpretation of data. JM contributed to the design of the study, to specific MR sequence protocols and to the analysis and interpretation of data. HW contributed to the acquisition of data. ME contributed to the acquisition and analysis of data. WF contributed to the conception of the study, to the acquisition of data, the analysis of data and the interpretation of data. IS contributed to the conception and to the acquisition of data. SG contributed to the creation of specific MR sequence protocols. HB contributed to specific MR sequence protocols. IS contributed to the conception of the study. HJB contributed to the acquisition of data. CB contributed to the conception and design of the study, to the acquisition of data and the analysis and interpretation of data. All authors read and approved the final draft.

Cooperators and coworkers in data post processing and analysis are permanently rising. At time of manuscript writing they are as follows: F. Birklein, M. Breimhorst, DC. Cheng, J. Ellermann, S. Faust, S. Göd, L. Heisterkamp, E. Kitzenmaier, K. König, S. König, TC. Mamisch, A. Reiner, D. Schoss, C. Tassler, C. Trattnig, F. Weber, S. Wuchenauer, A. Wunderlich and C. Würslin.

## Authors' information

Dr Uwe Schütz may also be contacted using his alternative email address: uwe.schuetz@uniklinik-ulm.de

## Pre-publication history

The pre-publication history for this paper can be accessed here:

http://www.biomedcentral.com/1741-7015/10/78/prepub
